# Digital Gene Expression Analysis to Screen Disease Resistance-Relevant Genes from Leaves of Herbaceous Peony (*Paeonia lactiflora* Pall.) Infected by *Botrytis cinerea*

**DOI:** 10.1371/journal.pone.0133305

**Published:** 2015-07-24

**Authors:** Saijie Gong, Zhaojun Hao, Jiasong Meng, Ding Liu, Mengran Wei, Jun Tao

**Affiliations:** Key Laboratory of Crop Genetics and Physiology of Jiangsu Province, College of Horticulture and Plant Protection, Yangzhou University, Yangzhou, Jiangsu, P.R. China; University of Nebraska-Lincoln, UNITED STATES

## Abstract

Herbaceous peony (*Paeonia lactiflora* Pall.) is a well-known traditional flower in China and is widely used for landscaping and garden greening due to its high ornamental value. However, disease spots usually appear after the flowering of the plant and may result in the withering of the plant in severe cases. This study examined the disease incidence in an herbaceous peony field in the Yangzhou region, Jiangsu Province. Based on morphological characteristics and molecular data, the disease in this area was identified as a gray mold caused by *Botrytis cinerea*. Based on previously obtained transcriptome data, eight libraries generated from two herbaceous peony cultivars ‘Zifengyu’ and ‘Dafugui’ with different susceptibilities to the disease were then analyzed using digital gene expression profiling (DGE). Thousands of differentially expressed genes (DEGs) were screened by comparing the eight samples, and these genes were annotated using the Gene ontology (GO) and Kyoto encyclopedia of genes and genomes (KEGG) database. The pathways related to plant-pathogen interaction, secondary metabolism synthesis and antioxidant system were concentrated, and 51, 76, and 13 disease resistance-relevant candidate genes were identified, respectively. The expression patterns of these candidate genes differed between the two cultivars: their expression of the disease-resistant cultivar ‘Zifengyu’ sharply increased during the early stages of infection, while it was relatively subdued in the disease-sensitive cultivar ‘Dafugui’. A selection of ten candidate genes was evaluated by quantitative real-time PCR (qRT-PCR) to validate the DGE data. These results revealed the transcriptional changes that took place during the interaction of herbaceous peony with *B*. *cinerea*, providing insight into the molecular mechanisms of host resistance to gray mold.

## Introduction

*Botrytis cinerea* Pers. (teleomorph: *Botryotinia fuckeliana* (de Bary) Fuck.), which leads to gray mold on various host plants [1], is considered one of the most important fungal plant pathogens worldwide [2]. As a necrotrophic fungus, it grows by relying on the nutrition of necrotic tissue after infecting hosts to trigger a hypersensitive response (HR); it always interferes with the physiological and biochemical functions of plants and may even cause the plants to wither and die [3–6]. Plants evolve several mechanisms to cope with *B*. *cinerea* stress, and these mechanisms are all achieved by induction of numerous disease resistance genes involved in various pathways [5–8]. A WRKY family gene that responds to *B*. *cinerea* infection, (*Solanum lycopersicum* defense-related WRKY1) *SlDRW1*, is significantly up-regulated by the defense response of tomato, and the silencing of this gene increases the severity of gray mold [8]. De Cremer et al. [6] show that genes involved in the phenylpropanoid pathway and terpenoid synthesis are transcribed in lettuce in response to a challenge by *B*. *cinerea*. The above studies reflect that disease resistance-relevant genes are highly effective against *B*. *cinerea* in host plants.

The continuous improvement of RNA-seq technology has provided a new approach for the functional genomics study of plants at the transcriptome level [9]. Digital gene expression profiling (DGE) is a new approach for transcriptome analysis that integrates high-throughput sequencing technology and high-performance computing analysis technology. It is mainly used to quantitatively study the gene expression profiles of specific tissues or cells in one species during specific biological processes, particularly concentrating on the study of gene expression differences at genome-wide level [10, 11]. DGE technology has lots of advantages, such as more accurate quantification, higher repeatability, wider detection range, and more reliable analysis. Recently, RNA-Seq has been widely used to study plants. However, few studies have examined the molecular mechanism of plant disease resistance via RNA-Seq and DGE technology. The use of DGE technology identifies numerous candidate genes that are specifically or commonly regulated at different stages of HR in an analysis of *Chenopodium amaranticolor* inoculated with *Tobacco mosaic virus* and *Cucumber mosaic virus*, highlighting the dynamic changes of the differentially expressed genes (DEGs) in the plant-pathogen interaction pathway [12]. DGE analysis is also used to compare healthy and infected tobacco plants at six sequential disease developmental stages and to identify thousands of DEGs and many biological processes involved in disease resistance response [13]. Analogously, Sun et al. [14] compared upland cotton and sea-island cotton infected with *Verticillium dahliae* and found that the hydroxycinnamoyl transferase gene (*HCT*) is up-regulated in upland cotton, whereas Phenylalanine Ammonialyase gene (*PAL*), 4-Coumarate:CoA ligase gene (*4CL*), Cinnamoyl Alcohol Dehydrogenase gene (*CAD*), Caffeoyl-CoA5-O-methyltransfenase gene (*CCoAOMT*), and caffeicacid-5-O-methyltransfenase gene (*COMT*) are up-regulated in sea-island cotton in the phenylalanine metabolism pathway. The successful uses of DGE technology described above provide a reference for studies of the molecular mechanism of other plants in response to *B*. *cinerea* infection.

Herbaceous peony (*Paeonia lactiflora* Pall.) is a well-known traditional flower in China with a reputation as “the minister of flowers”. It is widely used in landscaping and garden greening due to its large flower, elegant shape, gorgeous color and rich fragrance, and it has been extensively cultivated in more than 50 countries, such as the United States, France, the Netherlands, etc [15]. However, gray mold invariably develops on herbaceous peony plants grown in the Jiangsu and Zhejiang area of China, as evidenced by wizened and necrotic leaves, sunken and broken stems and brown and rotten petals [16]. These symptoms seriously affect the ornamental and commercial values of the plants. This study revealed that the herbaceous peony cultivars ‘Zifengyu’ and ‘Dafugui’ had been damaged by gray mold but that their resistance to the pathogen differed significantly. ‘Zifengyu’ grew well and showed few disease spots, while ‘Dafugui’ appeared weak and almost completely withered. The pathogen was identified as *B*. *cinerea* in both cases based on morphological characteristics and molecular data. At present, studies of the defense mechanism of herbaceous peony against *B*. *cinerea* are scarce and have mostly concentrated on the physiological and biochemical aspects of pathogenic fungus identification, biological characteristics, screening of resistant cultivars and chemical control [16]. Little is known about the disease resistance-relevant genes involved in the interaction between host and pathogen, which requires exploration into these mechanisms. In a previous study, transcriptome sequencing of leaves of two herbaceous peony cultivars ‘Zifengyu’ and ‘Dafugui’ harvested at four stages (from uninfected to severely infected, at May 25, June 15, July 5, and July 25, respectively) were performed via *de novo* RNA-seq technology to establish a database (Accession No. for library ‘Zifengyu’ SRS774325; Accession No. for library ‘Dafugui’ SRS774327). Based on this database, a DGE analysis of these two cultivars samples was conducted at four stages (the same as the transcriptome sequencing, from uninfected to severely infected, at May 25, June 15, July 5, and July 25, respectively) in order to identify the metabolic pathways and disease resistance-relevant genes of herbaceous peony plants that were involved in *B*. *cinerea* infection. These pathways and genes could provide a theoretical basis for comprehensively expounding the mechanism of herbaceous peony resistance to gray mold.

## Materials and Methods

### Plant materials

The herbaceous peony disease-resistant cultivar ‘Zifengyu’ and the disease-sensitive cultivar ‘Dafugui’ were examined in this study, which were planted in the germplasm repository of Horticulture and Plant Protection College, Yangzhou University, Jiangsu Province, P. R. China (32°30′N, 119°25′E). Because the disease severity gradually increased after flowering, the third and fourth node leaves at the top of the branch were taken at four stages from uninfected to severely infected: Stage 1 (S1, May 25), Stage 2 (S2, June 15), Stage 3 (S3, July 5), Stage 4 (S4, July 25); and S1 was the uninfected stage that taken as the control. One part of samples was used to identify the pathogenic fungus and determine physiological indexes, while the remainder was stored at -80°C in order to extract RNA for DGE and quantitative real-time PCR (qRT-PCR) analysis.

### Pathogen identification

The pathogenic fungus was isolated and purified via usual tissue isolation methods [17], and then isolated strains were cultured on potato sugar agar (PSA) plate medium to observe the colony morphology. After sporulation, the morphological characteristics were observed, photomicrographs of conidiophores and conidia were obtained, and the conidia size was measured. The healthy leaves were then inoculated in vivo and in vitro with the dominant pathogen with or without wounds, respectively. The tissue was again harvested after infection to isolate the pathogen from the leaves and verify that whether this pathogen was the same as the original inoculant. The pathogen morphology was preliminarily identified based on the references [18]. The genomic DNA of the pathogenic fungus was extracted with a fungal genomic DNA rapid extraction kit (Sangon Biotech (Shanghai) Co., Ltd.) for molecular identification, and the sequence was amplified with internal transcribed spacer (ITS) universal primers (forward primer ITS1: 5’-TCCGTAGGTGAACCTGCGG-3’, reverse primer ITS4: 5’-TCCTCCGCTTATTGATATGC-3’) [19]. The amplification products were separated via electrophoresis in an agarose gel, and the target fragment was then sequenced after purification. A comparative analysis of the rDNA-ITS sequence was performed using the Basic Local Alignment Search Tool (BLAST) tools from GenBank to identify the strain and its related species.

### Physiological indexes determinations

The relative conductivity and content of chlorophyll were measured with the conductivity meter method and the lixiviating method, respectively [20].

### RNA extraction and cDNA library construction for DGE

Equal quantities of three replications of each sample were mixed to extract the total RNA according to the CTAB extraction protocol [21], and the integrity of RNA was confirmed via Agilent 2100 Bioanaylzer and assessed by RNA Integrity Number (RIN). The eight samples were first treated with DNase I to degrade any possible DNA contamination, and the mRNA was then enriched by the oligo(dT) magnetic beads. Mixed with 5×fragmentation buffer (Illumina, USA), the mRNA was fragmented into short fragments at 94°C. The first strand of cDNA was synthesized by using random hexamer-primer; then Buffer, dNTPs, RNase H and DNA polymerase I were added to synthesize the second strand. The double strand cDNAs were purified with magnetic beads. End reparation and 3’-end single nucleotide A (adenine) addition were then performed. After that, sequencing adaptors were ligated to the fragments. Finally, the fragments were enriched by polymerase chain reaction (PCR) amplification to construct cDNA libraries. During the quality control (QC) step, Agilent 2100 Bioanaylzer and ABI StepOnePlus Real-Time PCR System were used to qualify and quantify the sample libraries, which were ready for sequencing when qualified.

### Sequencing and treatment of sequence data

The eight cDNA libraries were sequenced using Sequencing by Synthesis (SBS) method via Illumina HiSeq 2000 platform at the Beijing Genomics Institute (Shenzhen, China), and the read lengths were 50 bp. Original data that defined as raw reads were produced, and the clean reads were then obtained after removing reads with adaptor sequences, reads in which the percentage of unknown bases was >10% and reads in which the low quality base (base with quality value≤5) was >50%. The unigenes previously generated from the *de novo* transcriptome sequencing of ‘Zifengyu’ and ‘Dafugui’ that performed by our research group served as the reference database to map the generated clean reads using SOAPaligner/SOAP2 [22]. Moreover, the composition of raw reads, the sequencing saturation and the distribution of reads on reference genes were analyzed to assess the sequence quality. In addition, the gene expression level was calculated using the reads per kb per million reads (RPKM) method [23] based on the numbers of reads uniquely mapped to the specific gene and the total number of uniquely mapped reads in the sample. To evaluate the normality of the DGE data, the distribution of genes coverage (the percentage of a gene covered by reads) was also analyzed.

### Screening of DEGs and functional enrichment analysis

A strict algorithm was developed to screen DEGs between two samples [24]. The hypothesis was statistically tested using False Discovery Rate (FDR) control to determine the p-value threshold. Moreover, the fold change of the gene between different samples was calculated according to the expression of gene (RPKM value). The DEGs were defined as genes with the FDR ≤0.001 and the expression change exceeding 2-fold (**|**log_2_Foldchange|≥1) between two samples. A Gene Ontology (GO) functional enrichment analysis and a Kyoto Encyclopedia of Genes and Genomes (KEGG) pathway enrichment analysis of screened DEGs were performed to understand their biological function and involvement in metabolic pathways. The GO functional enrichment analysis was performed first by mapping genes to the GO database (http://www.geneontology.org/). The Blast2GO software and the WEGO software [25] were then used to obtain the GO annotation information and the GO functional classification of all DEGs. Using the KEGG public database [26], the significantly enriched metabolic pathways and signal transduction pathways in DEGs were identified by comparing the KEGG pathway enrichment analysis with the whole genome background.

### Expression pattern analysis of candidate DEGs

Taking | log_2_Foldchange |≥3 as the new standard, the candidate DEGs were screened again and classified according to their biological function. Furthermore, a cluster analysis of the log_2_ value of candidate DEGs fold change was performed with the cluster and Java Treeview software [27, 28]. Expression differences were shown in different colors, with red indicating up-regulation and green indicating down-regulation.

### Gene expression analysis by qRT-PCR

To determine the expression levels of selected candidate genes, qRT-PCR analysis was performed with three biological replications of each sample via a CFX96 Real-Time System (Bio-Rad, USA). The specific methods were referred to Zhao et al [29]. Three replications of RNA samples were used as templates for reverse transcription with PrimeScript RT reagent Kit With gDNA Eraser (TaKaRa, Japan). *P*. *lactiflora* Actin (JN105299) had been used as an internal control in this study [30]. Gene-specific primers were designed using PRIMER5.0 software and listed in S1 Table 2 μL of the cDNAs of each sample were used for ordinary PCR to test the amplification specificity of the corresponding primer pairs. qRT-PCR was performed using the SYBR Premix Ex Taq (Perfect Real Time) (TaKaRa, Japan). The amplification system consisted of an initial denaturation of 95°C/30 s, followed by 40 cycles of 95°C/5 s, 51°C/30 s, 72°C/30 s. Gene relative expression levels were calculated by the 2^-∆∆Ct^ comparative threshold cycle (Ct) method [31]. The Ct values of the triplicate reactions were gathered using the Bio-Rad CFX Manager V1.6.541.1028 software.

## Results

### Symptoms of herbaceous peony gray mold

**Fig 1:**
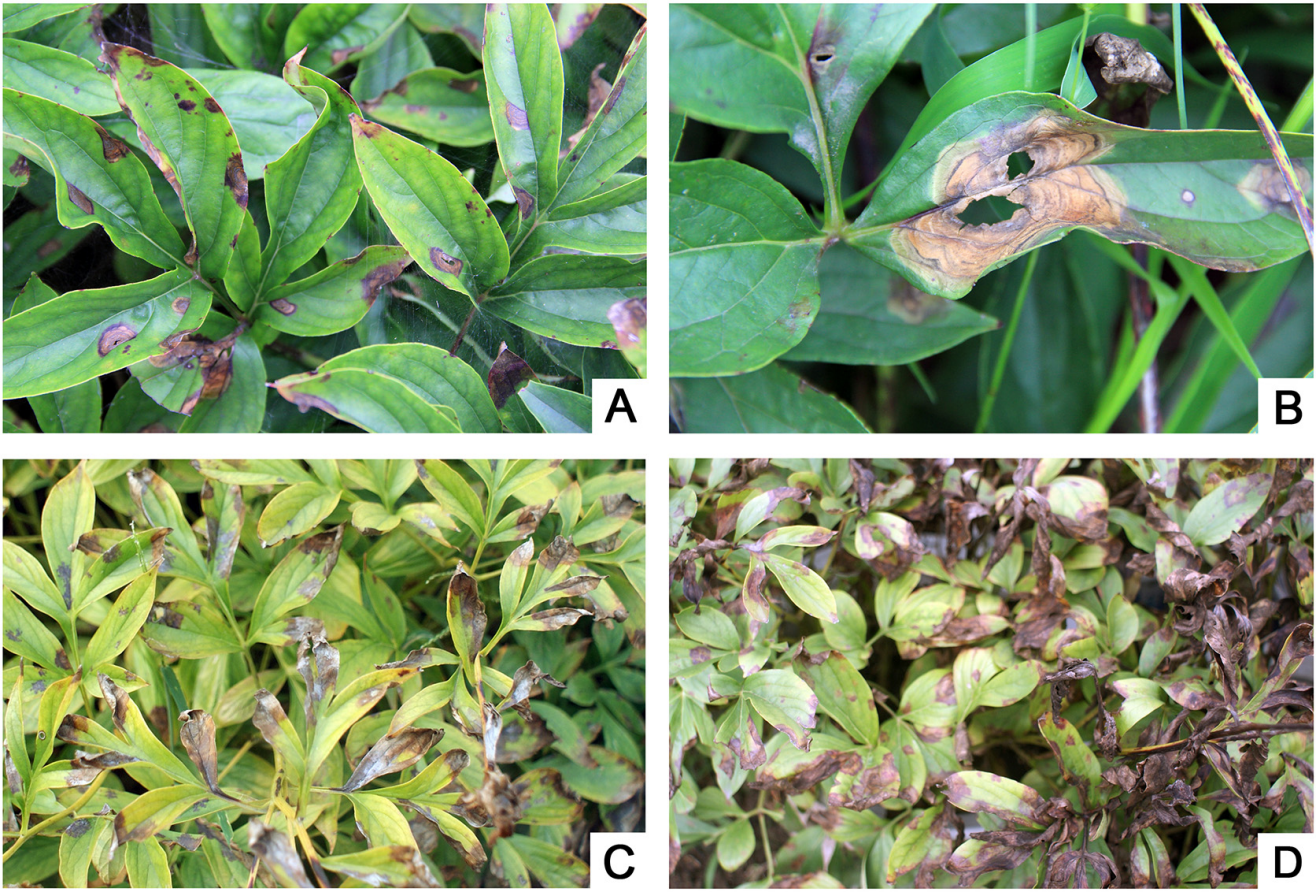
Symptoms of herbaceous peony gray mold. (A) Early symptoms of the disease on the leaf margin or tip. (B) The spots extended continuously to the interior of the leaf. (C) The gray molds grew out upon the infected positions due to high humidity. (D) When the humidity decreased, the infected positions wizened and turned brown or yellow.

The early symptoms of the disease occurred at early June were usually dark green water-soaked spots on the leaf margin or tip, which later extended continuously to the interior of the leaf with elliptic or irregularly shaped spots and round striae (Fig 1A and 1B). Brown rot was due to high humidity, while the gray molds grew on the infected positions (Fig 1C); when the humidity decreased, the infected positions wizened and turned brown or yellow (Fig 1D).

### Morphological characteristics of the pathogen

**Fig 2:**
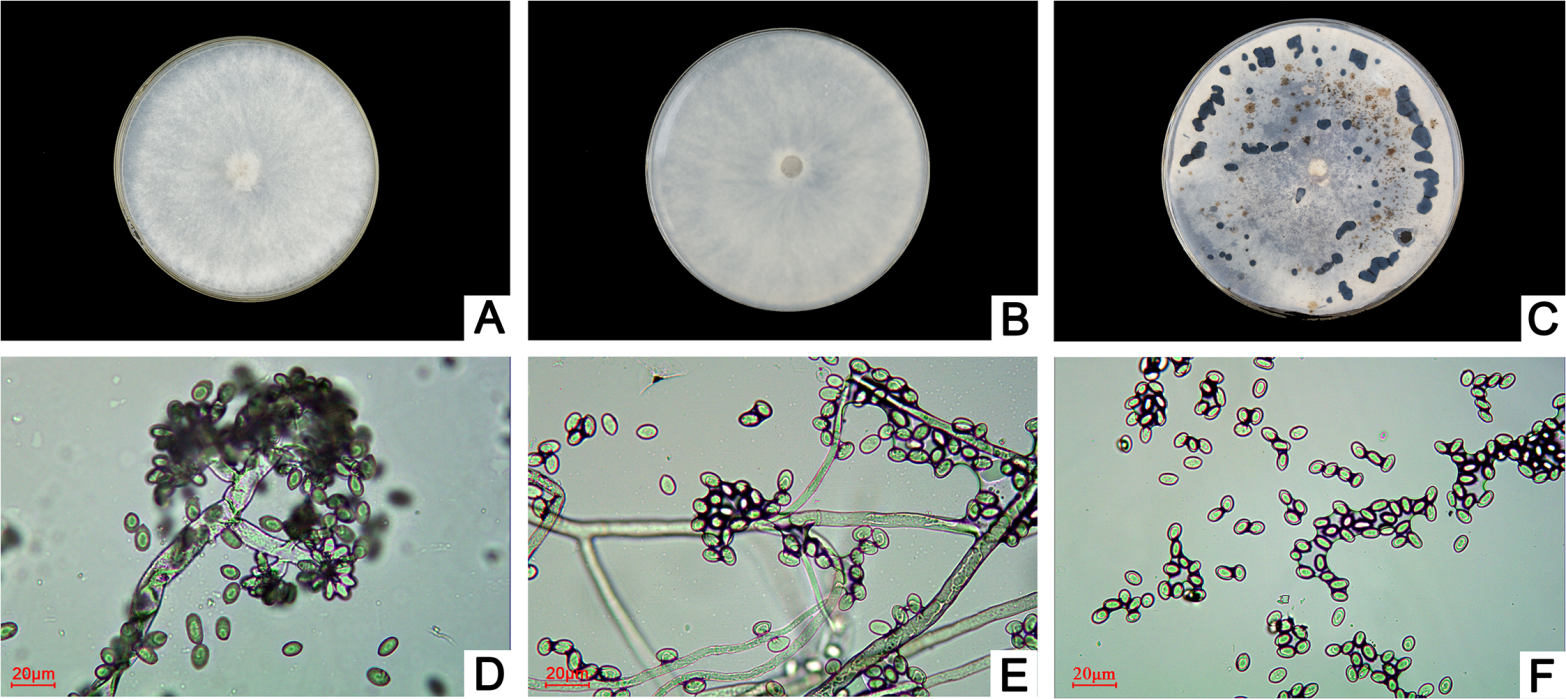
Morphological characteristics of the pathogen. (A and B) The front and back of the colony morphology on PSA plate medium. (C) Sclerotia morphology on PSA. (D-F) Conidiophore and conidia morphology on PSA.

The colonies on PSA, which appeared as white sparse villous mycelia early on, rapidly grew in the radial direction and covered the plate within 4 days (Fig 2A and 2B). Later, the colonies formed spores; conidiophores were scattered or tufty, erect in shape and gray or pale brown in color; the tops of conidiophores consisted of 1~2 branches, and the terminal branch expanded with dense small sporophores, while a large number of conidia generated grape spikes; the conidia were ovoid or elliptical in shape, colorless or grayish brown in color, and 7~14μm×4~9μm in size (Fig 2D–2F). As the aerial hyphae deepened in color, the mycelia gradually flocculated; approximately 10 days later, black circular sclerotia dispersed at the edge of the medium, and basal or half buried sclerotia then appeared on the colony surface, often ultimately gathering as large irregular or globular groups (Fig 2C). The above morphological characteristics of the pathogen were similar to *Botrytis*.

### Determination of pathogenicity and host range

**Fig 3:**
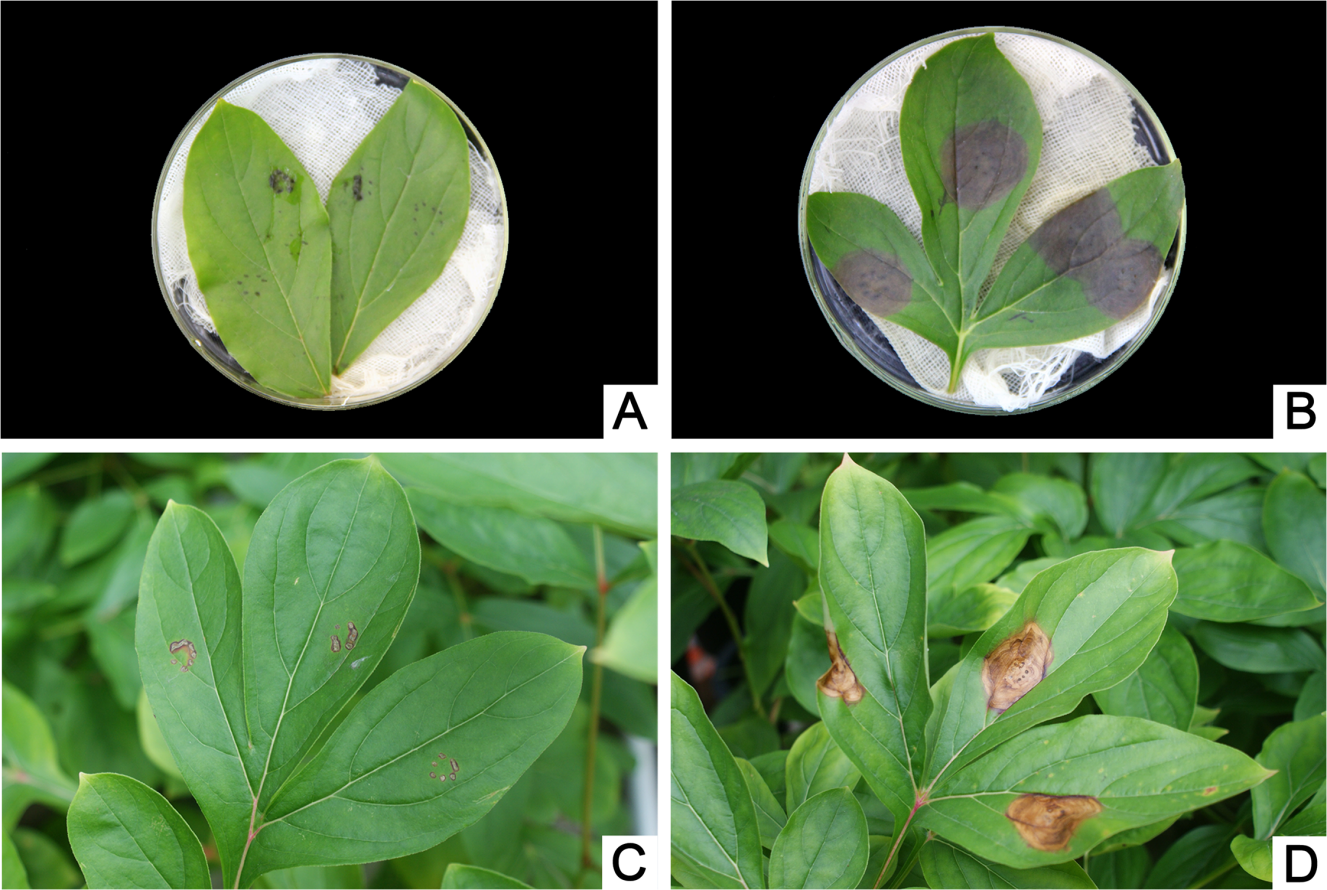
Infection status of the mock- and pathogen-inoculated herbaceous peony leaves with wounds. (A and C) Mock inoculation with wounds. (B and D) Inoculation with the pathogen with wounds. (A and B) Mock- and pathogen-inoculated leaves in vitro. (C and D) Mock- and pathogen-inoculated leaves in vivo.

When the leaves with wounds were inoculated with the pathogen in vitro and in vivo, respectively, taupe or tawny spots formed and extended continuously (Fig 3B and 3D), while no spots were observed in the mock inoculation (Fig 3A and 3C). Inoculation without wounds also resulted in infection with spots, but it was less severe compared with inoculation with wound. (Table 1). The infected leaves were isolated again after inoculation, and the obtained strain was the same as the inoculated one. According to Koch’s Rule, the isolated strain was identified as the pathogenic fungus of herbaceous peony gray mold. Relevant reports indicated that the pathogens of herbaceous peony gray mold included two species, *B*. *cinerea* and *Botrytis paeoniae* [32–34]. Thus, different plants were inoculated with the strain in order to determine the specific species of the dominant pathogen. The results shown in Table 1 revealed that the pathogen could infect a wide range of hosts in addition to herbaceous peony, such as gardenia (*Gardenia jasminoides*), Chinese rose (*Rosa chinensis*), camellia (*Camellia japonica*), tomato (*Lycopersicon esculentum*), green pepper (*Capsicum annuum* var. *grossum*), cucumber (*Cucumis sativus*) and Chinese cabbage (*Brassica pekinensis*). Because all selected host plants were infected with *B*. *cinerea* and not *B*. *paeoniae* [19], the isolated pathogen was believed to be *B*. *cinerea*.

**Table 1:** Determination of host range of the pathogen.

Host	Herbaceous peony (*Paeonia lactiflora* Pall.)	Gardenia (*Gardenia jasminoides*)	Chinese rose (*Rosa chinensis*)	Camellia (*Camellia japonica*)	Tomato (*Lycopersicon esculentum*)	Green pepper (*Capsicum annuum* var. *grossum*)	Cucumber (*Cucumis sativus*)	Chinese cabbage (*Brassica pekinensis*)
Inoculation with wounds	++++^a^	++++	+++^b^	+++	++++	+++	++^c^	++
Inoculation without wounds	++++	++++	+++	+++	++++	+^d^	+	+
Mock inoculation	—^e^	—	—	—	—	—	—	—

^a^ represents that the plants are infected after two days and the disease spots extend rapidly.

^b^ represents that the plants are infected after three days and the disease spots extend a bit slow.

^c^ represents that the plants are infected after four days and the disease spots extend slowly.

^d^ represents that the plants are infected after five days and few disease spots appear.

^e^ represents that the plants are not infected.

### Pathogenic rDNA-ITS sequence analysis

The sequence of the isolated strain was amplified with ITS universal primers, followed by agarose gel electrophoresis detection, which yielded a clear band of approximately 500bp (S1 Fig). The resulting 511bp sequence was deposited in GenBank (accession No. KP256186). Using the BLAST tools, the homology of this sequence with ITS sequences related to the strain from GenBank was determined. This strain was 99% similar to *B*. *cinerea* (teleomorph: *B*. *fuckeliana*), which supported that the isolate was *B*. *cinerea*. Thus, the morphological characteristics, pathogenicity, host range and rDNA-ITS sequence analysis of the isolated strain identified the pathogenic fungus of herbaceous peony gray mold as *B*. *cinerea* [16, 18, 35].

### Physiological indexes determination

**Fig 4:**
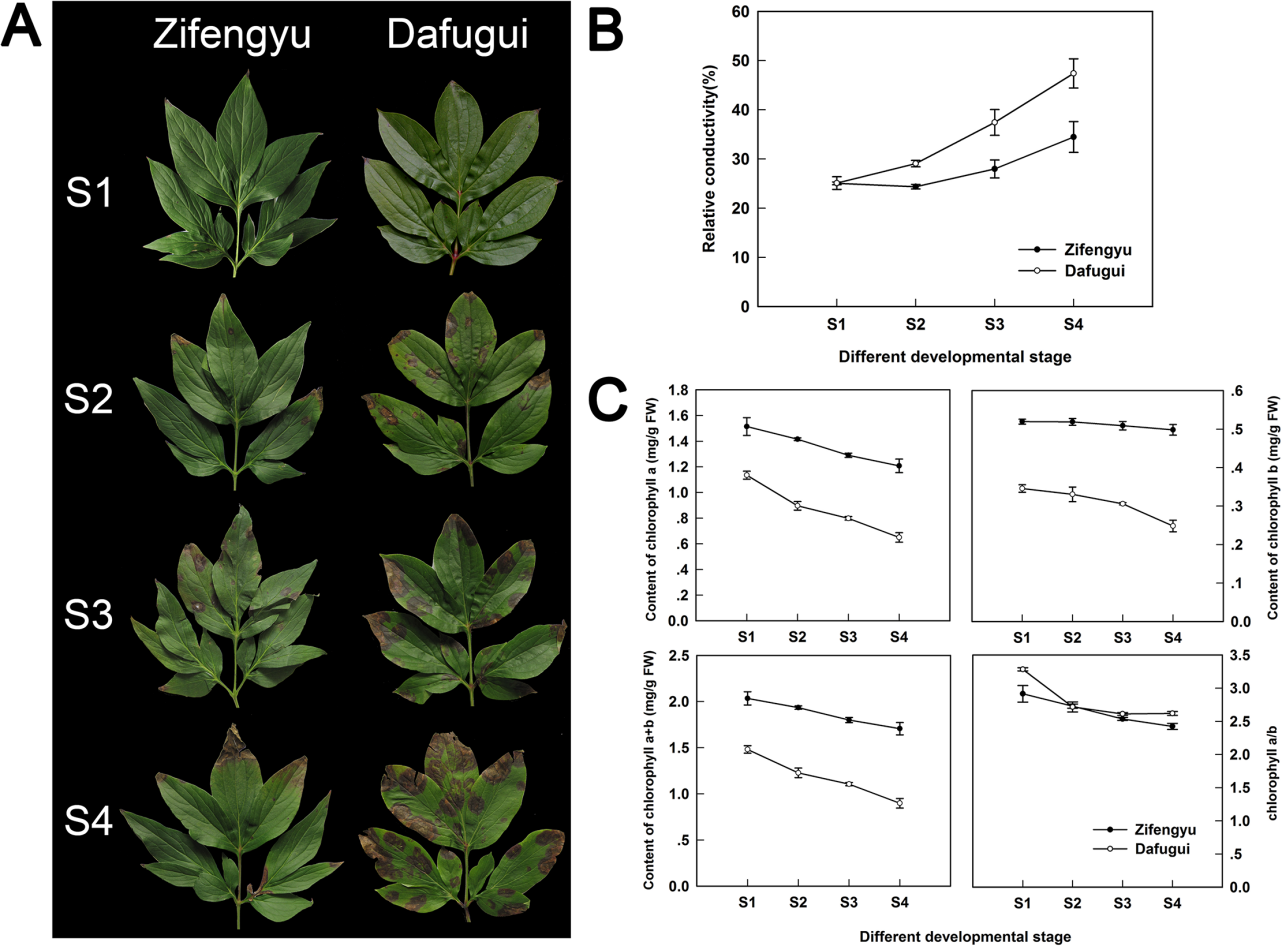
The infection status in the field and the physiological indexes changes of herbaceous peony. (A) The infection status of leaves of two herbaceous peony cultivars ‘Zifengyu’ and ‘Dafugui’ at four development stages. (B) The relative conductivity of leaves of ‘Zifengyu’ and ‘Dafugui’ at four development stages. (C) The content of chlorophyll a, b, a+b and the ratio of chlorophyll a content to chlorophyll b content of leaves of ‘Zifengyu’ and ‘Dafugui’ at four development stages. S1: May 25, S2: June 15, S3: July 5, S4: July 25.

The *B*. *cinerea* infection of herbaceous peony in the field worsened over time, and the infection was more severe in the ‘Dafugui’ variety than in the ‘Zifengyu’ variety (Fig 4A). Specifically, compared with ‘Zifengyu’, the infection time was earlier and the morbidity was higher in ‘Dafugui’; after infection, the spot spread faster in ‘Dafugui’ and thus the spot coverage was wider. At S4, ‘Zifengyu’ grew well and showed few disease spots, while ‘Dafugui’ appeared weak and almost completely withered. The relative conductivity and content of chlorophyll have been utilized as physiological indexes to reflect the disease resistance of plants in a large number of studies [36–38]. Thus, these two indexes were determined to verify the resistance of the herbaceous peony cultivars in this study. The relative conductivity of both ‘Zifengyu’ and ‘Dafugui’ increased with time, and ‘Dafugui’ was consistently higher than ‘Zifengyu’ (Fig 4B). Furthermore, the chlorophyll a, b, a+b contents in ‘Zifengyu’ and ‘Dafugui’ all decreased as development progressed, and the chlorophyll content of ‘Zifengyu’ consistently exceeded that of ‘Dafugui’ (Fig 4C). Although the ratio of the chlorophyll a content to the chlorophyll b content in the two cultivars also decreased overall, the ratio was slightly lower in the ‘Zifengyu’ variety than in the ‘Dafugui’ variety (Fig 4C). In conclusion, *B*. *cinerea* was less damaging to ‘Zifengyu’ than to ‘Dafugui’, i.e., “‘Zifengyu’ was more resistant to this pathogen than ‘Dafugui’, which was consistent with our field observations.

### DGE libraries sequencing and mapping

To identify the key resistance genes of herbaceous peony against *B*. *cinerea*, three replications of each sample were mixed equally to construct 8 cDNA libraries (‘Zifengyu’-S1 to ‘Zifengyu’-S4, ‘Dafugui’-S1 to ‘Dafugui’-S4) and subject to sequencing on an Illumina HiSeq 2000 platform. After removing low quality reads (including adaptor sequences or >10% uncertain bases), clean reads were obtained from eight libraries of ‘Zifengyu’ and ‘Dafugui’ (Table 2). The numbers of total mapped reads, perfect match, unique match, etc. were listed in Table 2. The proportions of clean raw reads all exceeded 99.20% in eight libraries (S2 Fig), which demonstrated that each sequencing library was of high quality and suitable for analysis in the next step.

**Table 2:** Summary of read mapping.

Sample ID	Total Reads	Total Base Pairs	Total Mapped Reads	Perfect Match	< = 2bpMismatch	Unique Match	Multi-position Match	Total Unmapped Reads
‘Zifengyu’-S1	12,660,873 (100.00%)	620,382,777 (100.00%)	9,975,444 (78.79%)	8,148,608 (64.36%)	1,826,836 (14.43%)	7,297,364 (57.64%)	2,678,080 (21.15%)	2,685,429 (21.21%)
‘Zifengyu’-S2	11,725,244 (100.00%)	574,536,956 (100.00%)	9,306,025 (79.37%)	7,550,470 (64.39%)	1,755,555 (14.97%)	7,642,074 (65.18%)	1,663,951 (14.19%)	2,419,219 (20.63%)
‘Zifengyu’-S3	11,976,718 (100.00%)	586,859,182 (100.00%)	9,027,136 (75.37%)	7,049,233 (58.86%)	1,977,903 (16.51%)	7,355,314 (61.41%)	1,671,822 (13.96%)	2,949,582 (24.63%)
‘Zifengyu’-S4	12,031,961 (100.00%)	589,566,089 (100.00%)	8,644,485 (71.85%)	6,622,238 (55.04%)	2,022,247 (16.81%)	7,226,805 (60.06%)	1,417,680 (11.78%)	3,387,476 (28.15%)
‘Dafugui’-S1	11,813,152 (100.00%)	578,844,448 (100.00%)	8,471,599 (71.71%)	6,561,507 (55.54%)	1,910,092 (16.17%)	6,329,400 (53.58%)	2,142,199 (18.13%)	3,341,553 (28.29%)
‘Dafugui’-S2	11,680,333 (100.00%)	572,336,317 (100.00%)	9,402,288 (80.50%)	7,312,040 (62.60%)	2,090,248 (17.90%)	7,887,030 (67.52%)	1,515,258 (12.97%)	2,278,045 (19.50%)
‘Dafugui’-S3	12,583,845 (100.00%)	616,608,405 (100.00%)	9,433,460 (74.96%)	7,220,166 (57.38%)	2,213,294 (17.59%)	7,879,255 (62.61%)	1,554,205 (12.35%)	3,150,385 (25.04%)
‘Dafugui’-S4	11,811,330 (100.00%)	578,755,170 (100.00%)	8,481,551 (71.81%)	6,446,867 (54.58%)	2,034,684 (17.23%)	7,127,034 (60.34%)	1,354,517 (11.47%)	3,329,779 (28.19%)

The data from the eight samples were mapped to the unigenes previously generated from *de novo* transcriptome sequencing by our research group (All RNA-Seq data had been deposited in the NCBI Sequence Read Archive database). The results were shown in Table 2: the proportion of clean reads that mapped to the database from the two cultivars exceeded 70% in both cases. The proportions of the identified genes positively correlated with the amount of clean reads, but the number of detected genes tended to saturate when the reads reached 6 million (6M) (S3 Fig), which indicates that the analysis requirements for the sampling depth were met. In addition, this finding revealed that the reads in every position were relatively evenly distributed on the reference genes in each library (S4 Fig), implying that the mRNA fragmentation was sufficiently random. Overall, the results of the sequencing analysis outlined above were sufficient for the subsequent analysis.

### Screening and analysis of DEGs

**Fig 5:**
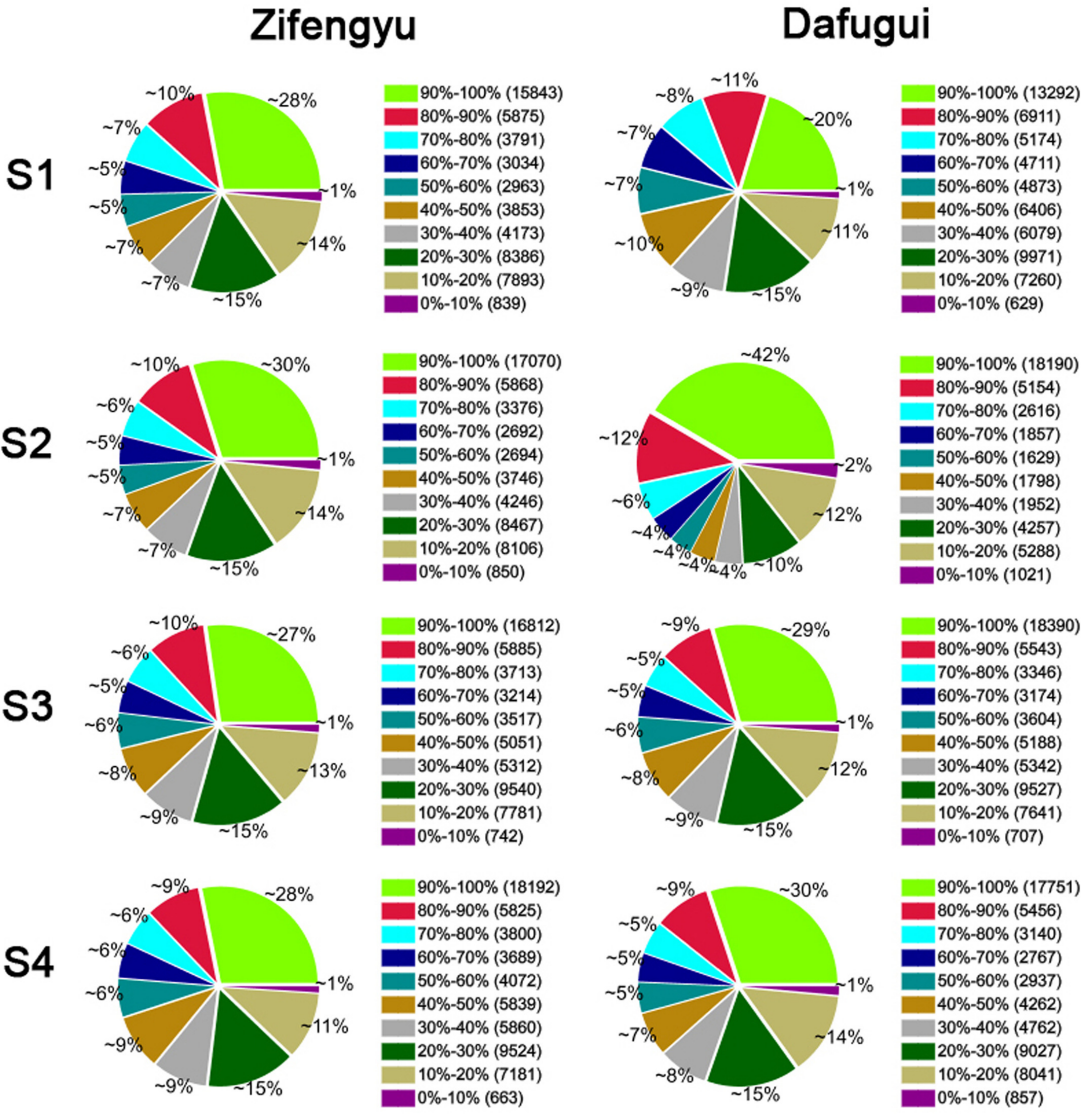
Distribution of gene coverage of each library. S1: May 25, S2: June 15, S3: July 5, S4: July 25.

To evaluate the normality of the DGE sequencing data, the distribution of gene coverage, i.e., the percentage of a gene covered by reads, was analyzed. In most samples, the proportion of gene coverage ≥90% ranged from 27% to 30%, while the proportion of gene coverage ≥70% ranged from 43% to 46%; only in ‘Dafugui’-S1, the proportions of gene coverage ≥90% and ≥70% were lower than the lowest level of the remaining samples, while the proportions in ‘Dafugui’-S2 were higher than the highest level of the remaining samples (Fig 5).

**Fig 6:**
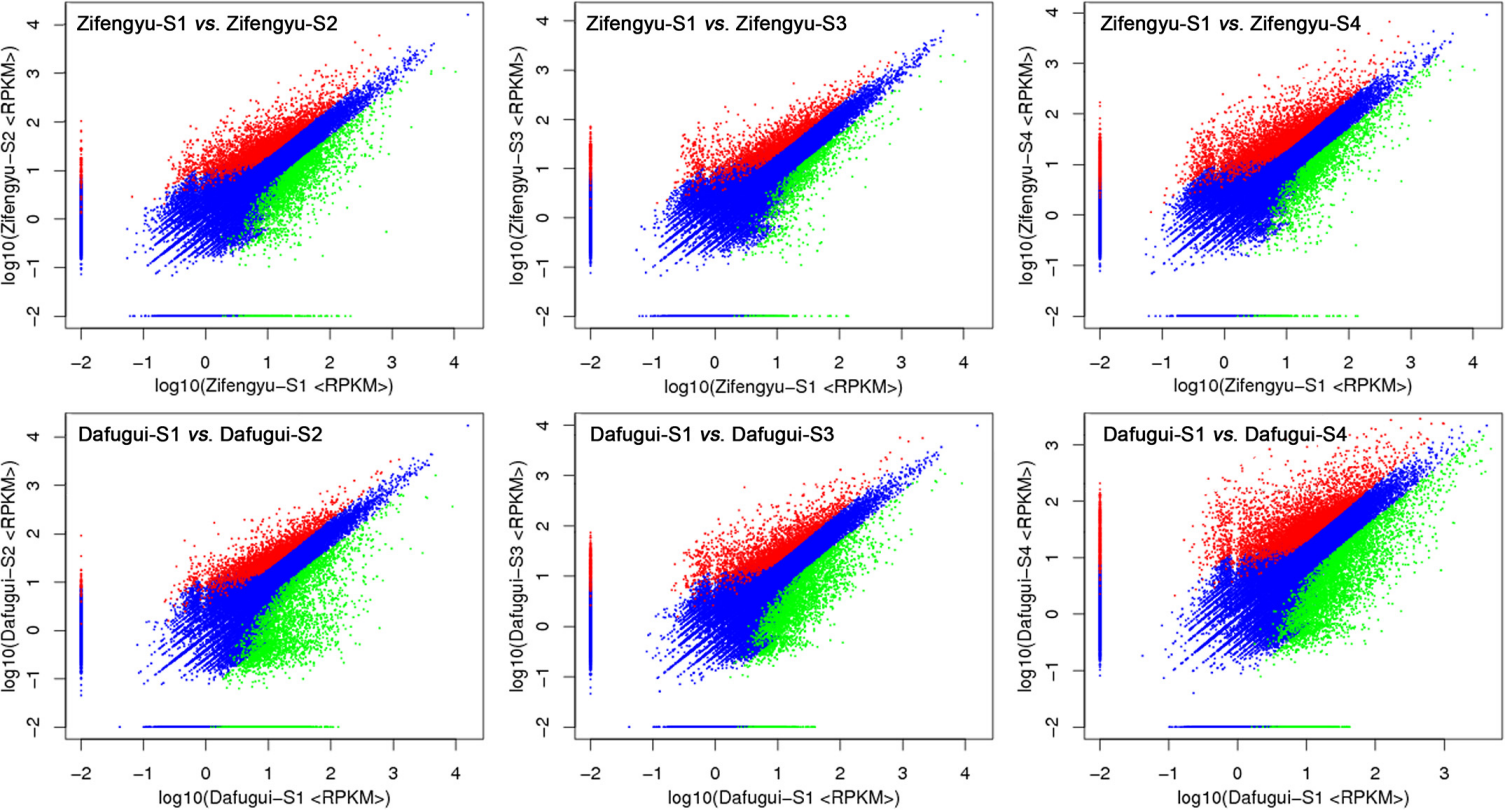
DEGs identified in each library contrast of the same cultivar at different stages. S1: May 25, S2: June 15, S3: July 5, S4: July 25. Red scatters indicate up-regulated DEGs, green scatters indicate down-regulated DEGs, and blue scatters indicate no difference DEGs in expression between the libraries.

To identify the candidate genes that conferred resistance to *B*. *cinerea* of herbaceous peony, the DEGs were screened by comparing the expression of different samples with a standard of FDR≤0.001 and | log_2_Foldchanges |≥1. First, the gene expressions of the two cultivars in S2, S3 and S4 were each compared to the expression of S1. Here, the number of DEGs in ‘Zifengyu’-S1 *vs*. ‘Zifengyu’-S2 reached 5876 (3433 up-regulated, 2443 down-regulated), and this number later decreased and then increased; the total number of DEGs in ‘Zifengyu’-S1 *vs*. ‘Zifengyu’-S4 reached a peak at 6843 (4916 up-regulated, 1927 down-regulated) (Fig 6). The overall trend for ‘Dafugui’ was consistent with that of ‘Zifengyu’, and the number of DEGs peaked at 10355 (5737 up-regulated, 4618 down-regulated) in ‘Dafugui’-S1 *vs*. ‘Dafugui’-S4 (Fig 6). Next, the gene expression levels in ‘Zifengyu’ and ‘Dafugui’ were compared at the same stage (taking ‘Zifengyu’ as the control). The total numbers of DEGs first increased and then decreased, and this number peaked at 4225 (2324 up-regulated, 1901 down-regulated) in ‘Zifengyu’-S3 *vs*. ‘Dafugui’-S3; however, the number of up-regulated genes (2370) in ‘Zifengyu’-S1 *vs*. ‘Dafugui’-S1 was highest among all the comparisons (S5 Fig).

### Functional enrichment analysis of DEGs

**Fig 7:**
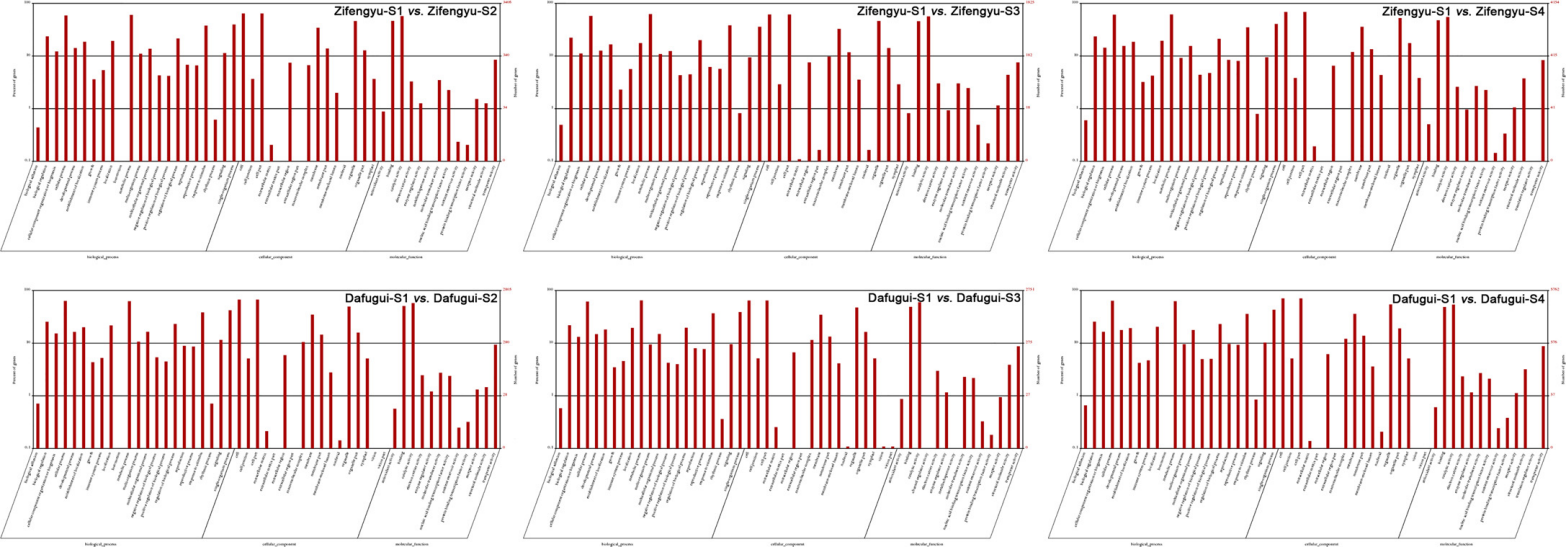
Go classifications of DEGs in each library contrast of the same cultivar at different stages. S1: May 25, S2: June 15, S3: July 5, S4: July 25. DEGs are annotated in three categories: biological process, cellular component and molecular function.

The GO functional enrichment analysis (p-value≤0.05) of the screened DEGs above identified some biological processes that were closely related to *B*. *cinerea-*induced stress. When comparing the samples in the same cultivar at different stages, the stress-relevant terms in ‘Zifengyu’-S1 *vs*. ‘Zifengyu’-S2 and ‘Dafugui’-S1 *vs*. ‘Dafugui’-S4 were enriched the most (S2 and S3 Tables). When comparing ‘Zifengyu’ and ‘Dafugui’ samples at the same stage, the “response to wounding”, “response to chitin” and “response to biotic stimulus” processes in ‘Zifengyu’-S4 *vs*. ‘Dafugui’-S4 were enriched the most (S4 Table). The GO functional classification revealed that the numbers of DEGs in ‘Zifengyu’-S1 *vs*. ‘Zifengyu’-S4 differed the most at different stages in ‘Zifengyu’ and involved 15 “cellular component” (primarily “cell” and “cell part”), 13 “molecular function” (primarily “catalytic activity” and “binding”) and 21 “biological process” ontologies (primarily “metabolic process” and “cellular process”); the equivalent numbers in ‘Dafugui’-S1 *vs*. ‘Dafugui’-S4 of ‘Dafugui’ involved 17 “cellular component”, 13 “molecular function” and 22“biological process” ontologies, and the most prevalent of these processes agreed with those identified in ‘Zifengyu’ (Fig 7). In the contrast to the two cultivars at the same stage, the highest number of DEGs was found in ‘Zifengyu’-S3 *vs*. ‘Dafugui’-S3, with 15 “cellular component”, 13 “molecular function” and 22 “biological process” ontologies, as listed in S6 Fig.

To further assess the biochemical metabolic and signal transduction pathways of DEGs, the significantly enriched pathways were identified by comparing them to the KEGG database. Here, we focused on the differences between ‘Zifengyu’-S1 *vs*. ‘Zifengyu’-S4 and ‘Dafugui’-S1 *vs*. ‘Dafugui’-S4, which yielded the highest number of DEGs. When comparing ‘Zifengyu’-S4 to ‘Zifengyu’-S1, 3067 DEGs were annotated to 126 metabolic pathways, 39 of which met the Q-value≤0.05 requirement (S5 Table). Among these pathways, the “metabolic pathways” included the most DEGs (891, 29.05%, ko01100), followed by the “biosynthesis of secondary metabolites” (559, 18.23%, ko01110), “plant-pathogen interaction” (207, 6.75%, ko04626), “plant hormone signal transduction” (176, 5.74%, ko04075) and “Ribosome” (149, 4.86%, ko03010) etc. The comparison of ‘Dafugui’-S4 to ‘Dafugui’-S1 annotated 4226 DEGs to 128 pathways, 37 of which met the Q-value≤0.05 requirement (S6 Table). Most of these DEGs clustered in the “metabolic pathways” category (1127, 26.67%, ko01100), followed by the “biosynthesis of secondary metabolites” (682, 16.14%, ko01110), “plant-pathogen interaction” (275, 6.51%, ko04626), “plant hormone signal transduction” (229, 5.42%, ko04075) and “Phenylpropanoid biosynthesis” (121, 2.86%, ko00940) etc. Some pathways closely related to *B*. *cinerea*-induced stress were significantly enriched, such as the “plant-pathogen interaction”, “Phenylpropanoid biosynthesis”, “Peroxisome” and “biosynthesis of secondary metabolites”, which warranted further study.

### Expression pattern analysis of candidate DEGs

**Fig 8:**
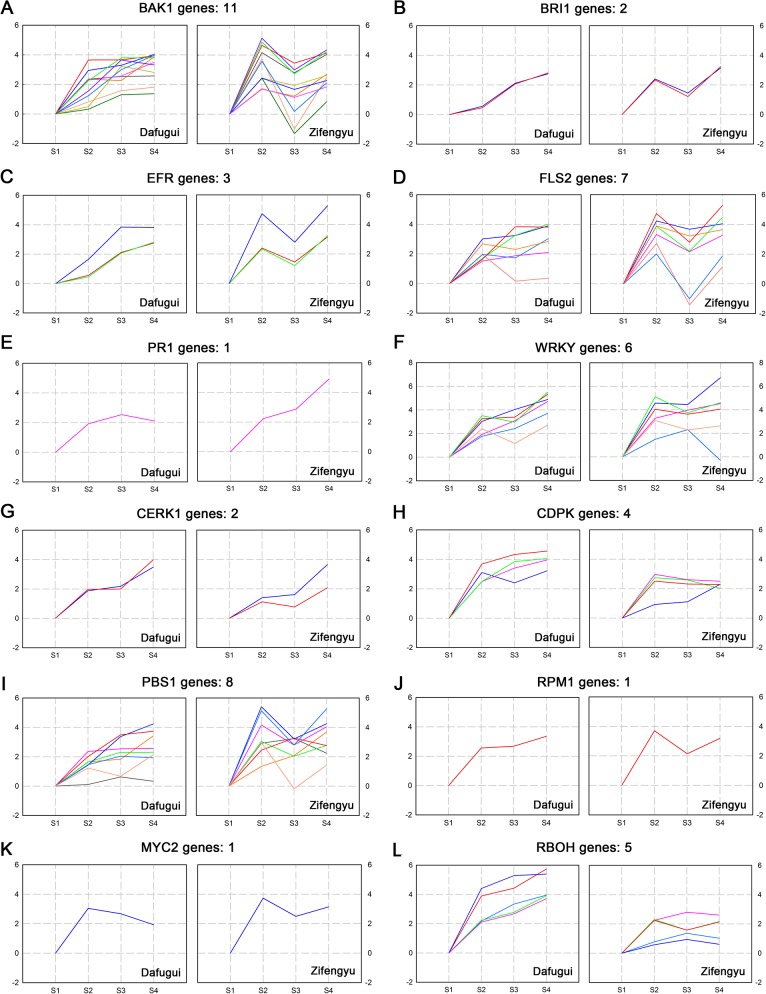
Expression pattern of candidate disease resistance-relevant genes involved in plant-pathogen interaction. Disease resistance-relevant genes involved in plant-pathogen interaction of ‘Dafugui’ and ‘Zifengyu’ at S1, S2, S3 and S4 stages were clustered based on their relative expression levels. S1: May 25, S2: June 15, S3: July 5, S4: July 25. The number of genes in each cluster is specified above each map. The Y axis represents normalized log_2_ value of gene expression levels. The X axis represents the different stages of infection.

The KEGG pathway enrichment analysis identified many disease resistance-relevant genes in the “plant-pathogen interaction” category that involved various resistance pathways. To screen the key genes closely related to resistance, the criterion was raised to | log_2_Foldchanges |≥3, and the screened DEGs were then categorized according to their biological functions. Specifically, eleven brassinosteroid insensitive 1-associated receptor kinase 1 (*BAK1*) genes, two brassinosteroid insensitive 1 (*BRI1*) genes, three elongation factor Tu receptor (*EFR*) genes, seven flagellin-sensitive 2 (*FLS2*) genes, one pathogenesis-related protein 1 (*PR1*) gene, six *WRKY* genes, two chitin elicitor receptor kinase 1 (*CERK1*) genes, four calcium-dependent protein kinase (*CDPK*) genes, eight *PBS1* genes, one *RPM1* gene, one *MYC2* gene and five respiratory burst oxidase homologue (*RBOH*) genes were identified. The gene expressions and annotations were all listed in S7 Table. Fig 8 clearly showed the different expression patterns of ‘Zifengyu’ and ‘Dafugui’ at four stages. For ‘Dafugui’, the expressions of DEGs continuously increased over time and peaked at S4, while the changes were more abrupt in ‘Zifengyu’ at S2, suddenly decreased at S3 and then increased again at S4. This finding revealed that the expression of resistance-relevant genes was strongly induced in the disease-resistant cultivar ‘Zifengyu’ during the early infection phases, while the expression of these genes was gradually induced in the disease-sensitive cultivar ‘Dafugui’, which allowed the fungus to damage ‘Dafugui’ more than ‘Zifengyu’ at the same stage.

**Fig 9:**
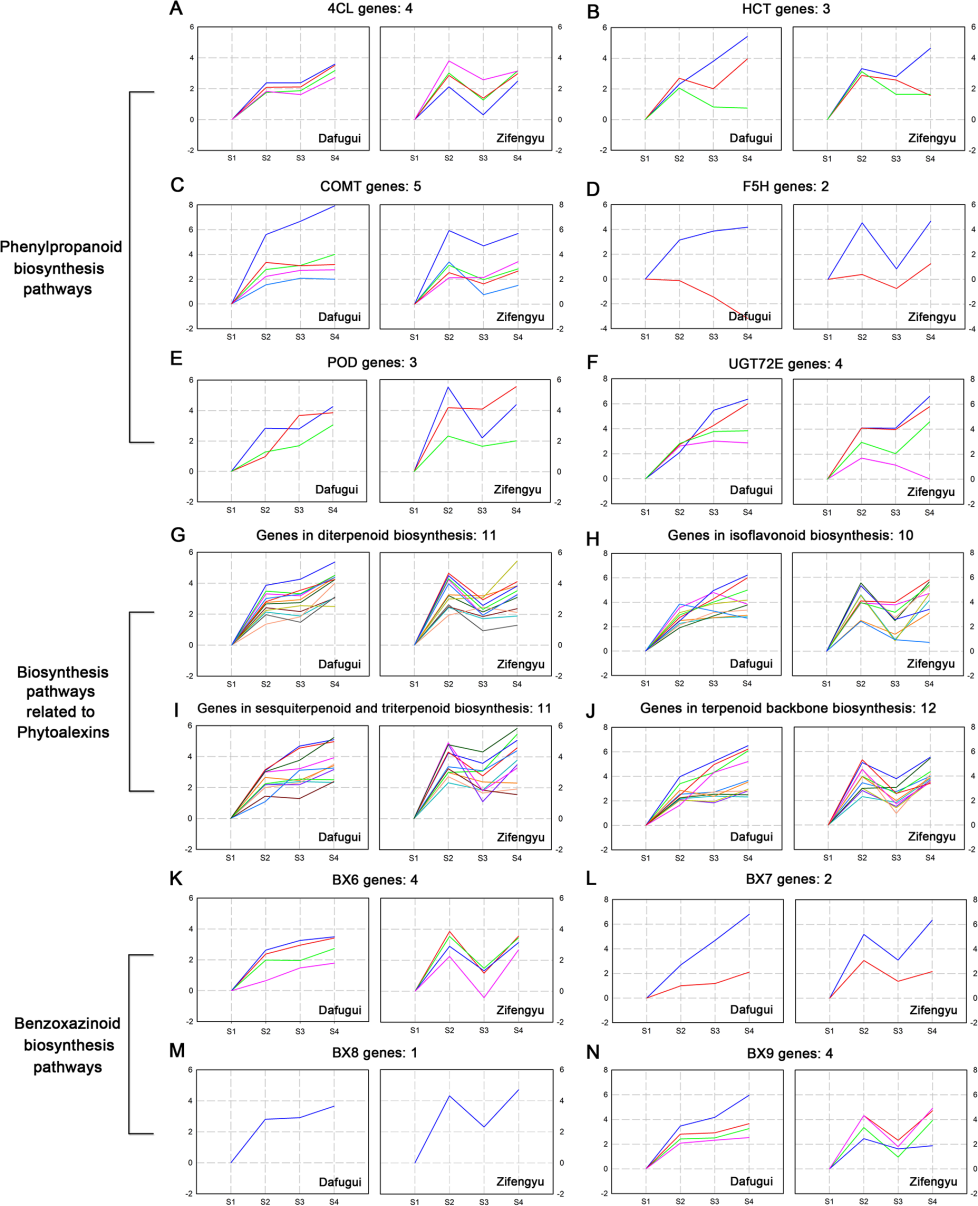
Expression pattern of candidate disease resistance-relevant genes involved in secondary metabolism. Disease resistance-relevant genes involved in secondary metabolism of ‘Dafugui’ and ‘Zifengyu’ at S1, S2, S3 and S4 stages were clustered based on their relative expression levels. S1: May 25, S2: June 15, S3: July 5, S4: July 25. (A-F) Candidate DEGs involved in phenylpropanoid biosynthesis pathways. (G-J) Candidate DEGs involved in biosynthesis pathways related to phytoalexins. (K-N) Candidate DEGs involved in benzoxazinoid biosynthesis pathways. The number of genes in each cluster is specified above each map. The Y axis represents normalized log_2_ value of gene expression levels. The X axis represents the different stages of infection.

Secondary metabolites could be used as biochemical barriers to defend the plant against pathogenic infection, and they could also serve as signaling molecules in defense reactions [39]. Many disease resistance-relevant genes that participated in secondary metabolism were identified in the pathways. The same screening criterion (| log_2_Foldchanges |≥3) was applied, and the category and annotation of the genes were detailed in S8 Table. Specifically, four *4CL* genes, three *HCT* genes, five *COMT* genes, two ferulate 5-hydroxylase (*F5H*) genes, four UDP-glycosyltransferase 72E (*UGT72E*) genes and three peroxidase (*POD*) genes were identified in “Phenylpropanoid biosynthesis”; in addition, the biosynthesis pathways related to phytoalexin included eleven genes in “Diterpenoid biosynthesis”, ten genes in “Isoflavonoid biosynthesis”, eleven genes in “Sesquiterpenoid and triterpenoid biosynthesis” and twelve genes in “Terpenoid backbone biosynthesis” category; furthermore, four *BX6* genes, two *BX7* genes, one *BX8* gene and four *BX9* genes were identified in the “Benzoxazinoid biosynthesis” category. Fig 9 clearly indicated that the expression patterns of DEGs differed between the two cultivars: their expression consistently increased in the ‘Dafugui’ variety, while it fluctuated in the ‘Zifengyu’ variety. Therefore, the resistance-relevant genes involved in the secondary metabolism of the ‘Zifengyu’ variety also sharply increased during the early stages of infection, while this expression was relatively subdued in the ‘Dafugui’ variety.

**Fig 10:**
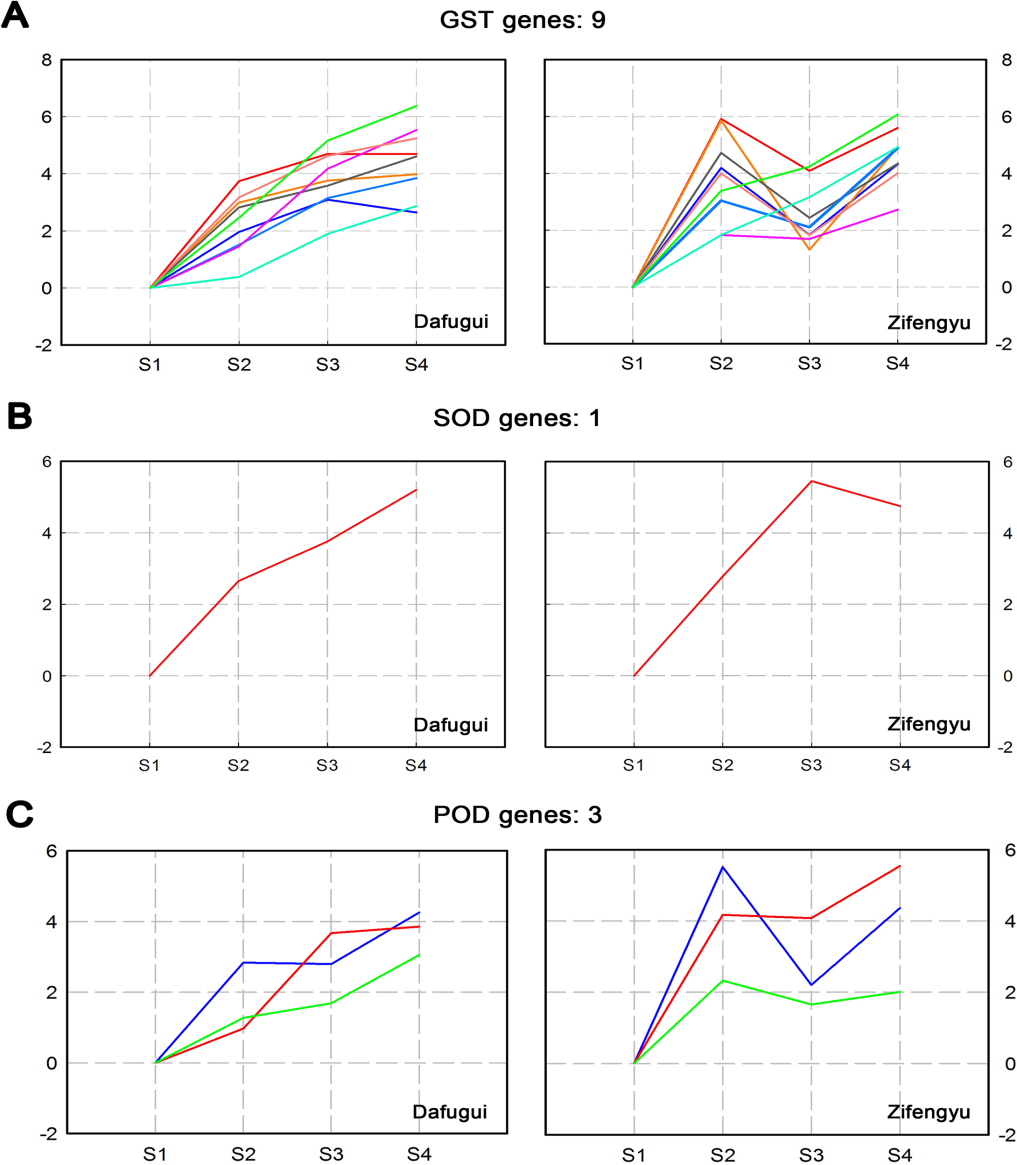
Expression pattern of candidate disease resistance-relevant genes involved in antioxidant system. Disease resistance-relevant genes involved in antioxidant system of ‘Dafugui’ and ‘Zifengyu’ at S1, S2, S3 and S4 stages were clustered based on their relative expression levels. S1: May 25, S2: June 15, S3: July 5, S4: July 25. The number of genes in each cluster is specified above each map. The Y axis represents normalized log_2_ value of gene expression levels. The X axis represents the different stages of infection.

In addition, several DEGs involved in the antioxidant system of plant were screened (S9 Table), which were responsible for the scavenging of ROS when plants suffered from pathogens. Specifically, one Superoxide Dismutase (*SOD*) gene, three *POD* genes, and nine glutathione S-transferase (*GST*) were identified. As in Fig 10, the gene expression patterns also differed between the two cultivars. The expression levels of genes encoded antioxidant enzyme of ‘Zifengyu’ were higher than those of ‘Dafugui’ at S2, which indicated more effective scavenging of ROS in ‘Zifengyu’ at early infection stages.

**Fig 11:**
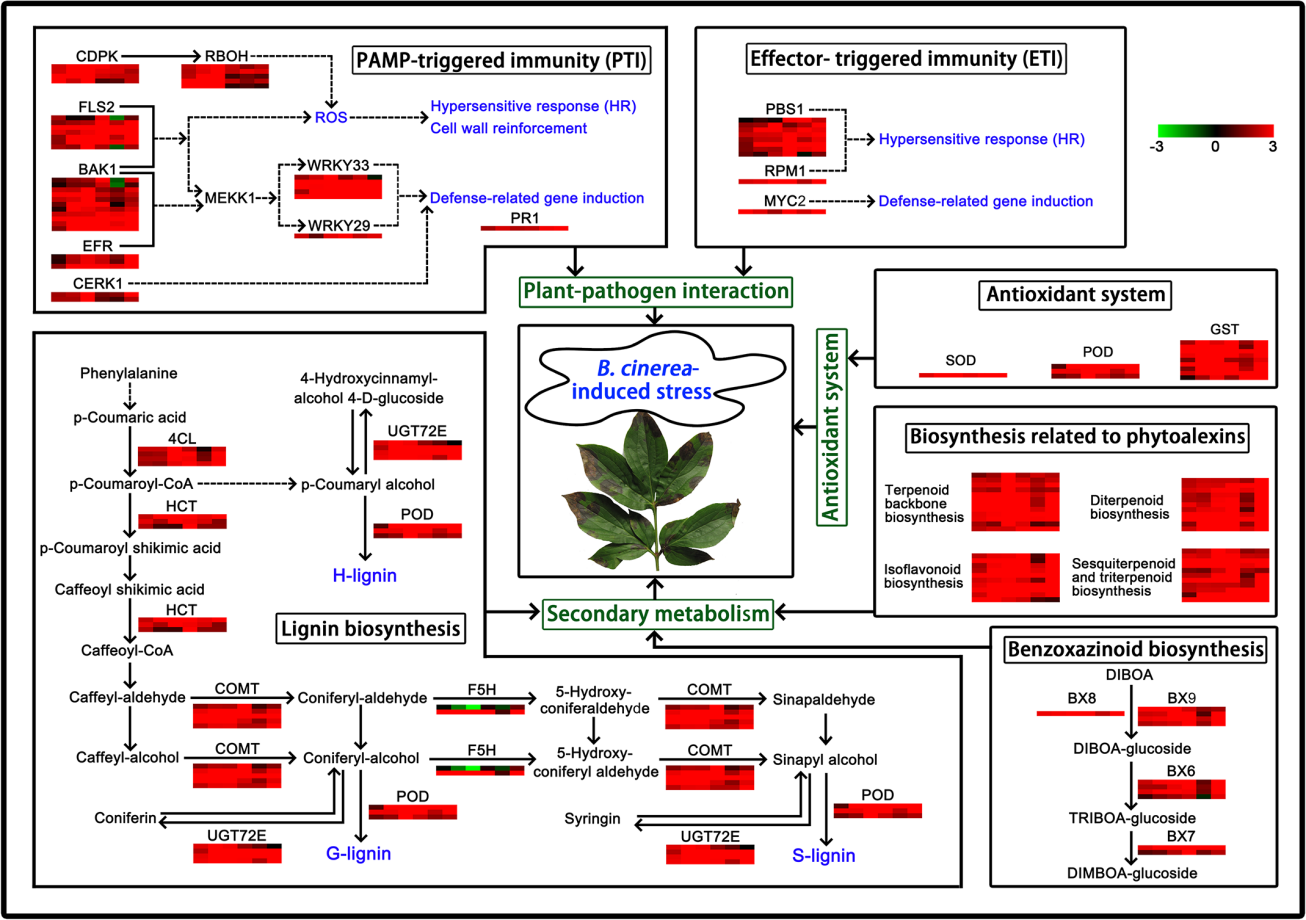
An overview of defense responses of herbaceous peony in response to *B*. *cinerea*-induced stress. *B*. *cinerea* infection induced defense responses involved in plant-pathogen interaction and secondary metabolism of herbaceous peony. CDPK, FLS2, BAK1, EFR, CERK1, RBOH, WRKY33, WRKY29 and PR1 are mainly involved in PAMP-triggered immunity (PTI), while PBS1, RPM1 and MYC2 are mainly involved in effector-triggered immunity (ETI). PTI and ETI are involved in plant-pathogen interaction. 4CL, HCT, COMT, F5H, POD and UGT72E are mainly involved in lignin biosynthesis. Diterpenoid biosynthesis, sesquiterpenoid and triterpenoid biosynthesis, terpenoid backbone biosynthesis and isoflavonoid biosynthesis are mainly involved in biosynthesis related to phytoalexins. BX6, BX7, BX8 and BX9 are mainly involved in benzoxazinoid biosynthesis. Lignin biosynthesis, biosynthesis related to phytoalexins and benzoxazinoid biosynthesis are involved in secondary metabolism. SOD, POD, GST are mainly involved in antioxidant system. The expression patterns of disease resistance-relevant genes are shown in coloured squares, from left to right, which represent the levels in ‘Dafugui’-S1 vs. ‘Dafugui’-S2, ‘Dafugui’-S1 vs. ‘Dafugui’-S3, ‘Dafugui’-S1 vs. ‘Dafugui’-S4, ‘Zifengyu’-S1 vs. ‘Zifengyu’-S2, ‘Zifengyu’-S1 vs. ‘Zifengyu’-S3 and ‘Zifengyu’-S1 vs. ‘Zifengyu’-S4, respectively. Red indicates up-regulation while green indicates down-regulation.

An overview of defense responses involved in the plant-pathogen interaction, secondary metabolism and antioxidant system was performed based on the cluster analysis of the candidate DEGs expression (Fig 11). The figure showed that the expression patterns of disease resistance-relevant genes in ‘Zifengyu’ and ‘Dafugui’ in response to *B*. *cinerea* differed based on the four stages, which explained the stronger disease resistance of ‘Zifengyu’ compared to ‘Dafugui’ along with the above conclusion.

### Expression analysis of candidate genes by qRT-PCR

**Fig 12:**
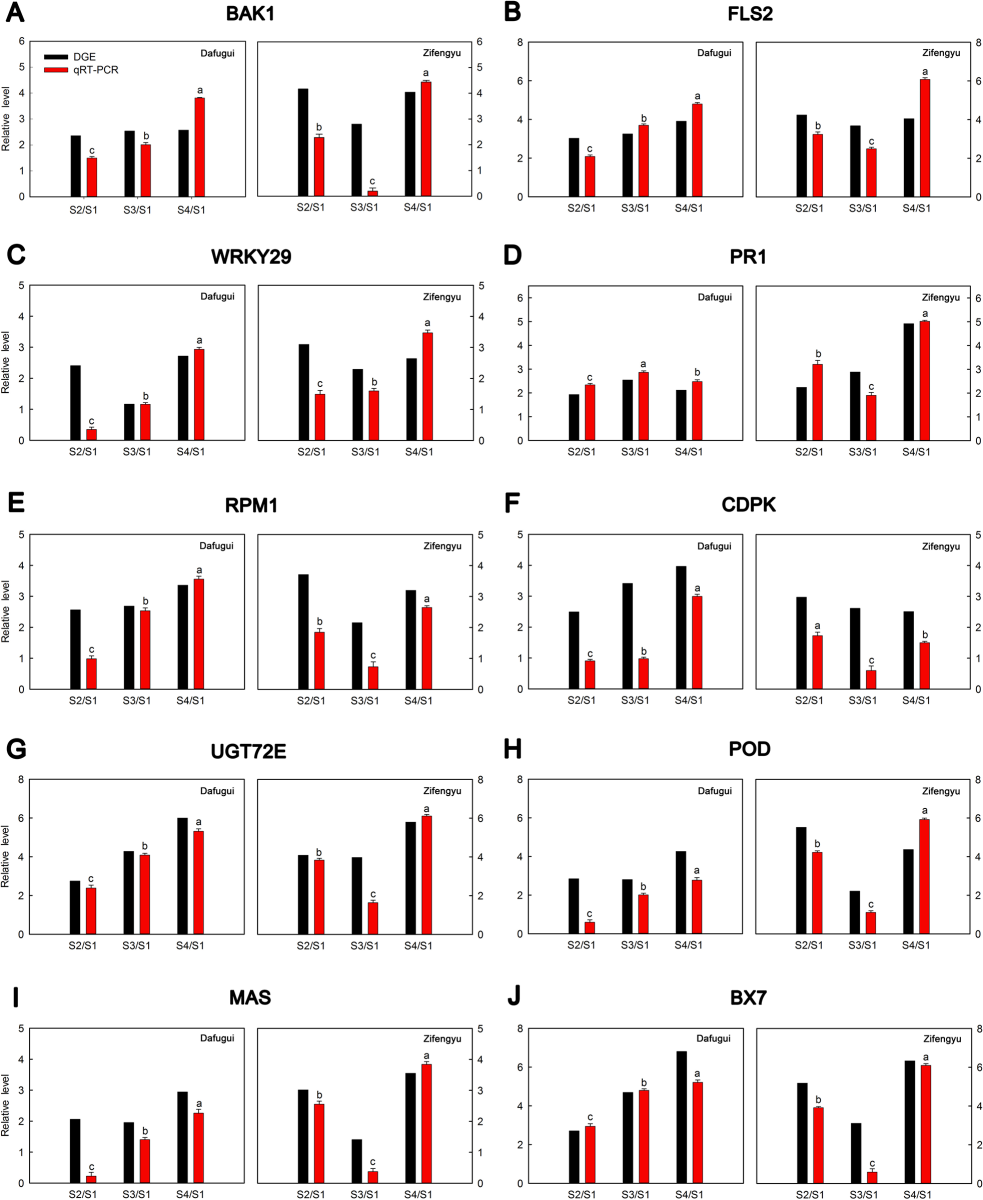
qRT-PCR validations of expression levels of candidate genes from DGE analysis. Expression levels by qRT-PCR of selected candidate genes of herbaceous peony cultivars ‘Dafugui’ and ‘Zifengyu’ were validated from the levels of DGE data. The corresponding genes is specified above each map. The Y axis represents normalized log_2_ value of gene expression levels. The X axis represents the comparisons of different stages. “S2/S1” indicates a comparison of gene expression levels between S1 and S2. “S3/S1” and “S4/S1” indicate analogous comparisons. S1: May 25, S2: June 15, S3: July 5, S4: July 25. Error bars represent the standard deviations of three biological replications, while the lowercase letters over the bars indicate significant differences (*P*<0.05).

To validate the results of DGE, expression levels of ten candidate genes including *BAK1*, *FLS2*, *PR1*, *WRKY29*, *CDPK*, *RPM1*, *UGT72E*, *POD*, momilactone-A synthase (*MAS*) and *BX7* were evaluated by qRT-PCR method. The data of results were listed in Fig 12. Overall, the relative expression profiling of the examined genes of ‘Dafugui’ and ‘Zifengyu’ in four stages (i.e., S1, S2, S3, S4) showed similar expression patterns as found in DGE profiles, which indicated a correspondence of the results from qRT-PCR analysis with the DGE sequencing analysis.

## Discussion

Plants always suffer from a variety of pathogens, such as bacteria, fungi and viruses. These pathogens always prevent the growth and development of plants and may even lead them to death. As a result of the long-term interaction and cooperative coevolution with the pathogens, plants gradually develop a series of complex and effective defense mechanisms [40–42]. These mechanisms not only include wax, cutin, phytohemagglutinin on the surface of the plant etc. that serve as physical and chemical barriers, but also contain a series of defense responses induced by pathogen infection, such as lignification of the infection site, activation of pathogenesis-related genes, and accumulation of phytoalexins. The difference between disease-resistant and disease-sensitive plants mainly depends on the timeliness with which the plant host recognizes pathogens, as well as the speed and effectiveness of the host defense mechanism excitation [43]. Disease-sensitive plants allow the pathogen to invade and eventually spread because the defense response is sluggish or weak [43]. In the natural environment, herbaceous peony is susceptible to the infestation of *B*. *cinerea*, which causes the overground parts to rot. Thus, a DGE analysis of the disease-resistant cultivar ‘Zifengyu’ and disease-sensitive cultivar ‘Dafugui’ was carried out in this study in an attempt to preliminarily clarify the molecular mechanism of herbaceous peony resistance to gray mold based on a combination of known plant disease resistance mechanisms.

### Pathogen identification of herbaceous peony gray mold

The pathogen that reportedly causes herbaceous peony gray mold differs by location. In the west, the pathogens *B*. *cinerea* and *B*. *paeoniae* reportedly cause this disease [32–34], while Lan [44] identifies the pathogen in China, such as in the cities of Nanjing and Shanghai, as *B*. *cinerea*. However, Yu [45] and Chen et al. [46] both identify *B*. *paeoniae* as the culprit of this disease in Luoyang and Sichuan, respectively. The two pathogens cause similar infection symptoms and morphological characteristics, but the sclerotia size and host range consistently differ. The sclerotia of *B*. *paeoniae* are 1.0~2.5 mm in size and rarely gather, while the sclerotia of *B*. *cinerea* are larger than 2.5 mm in size and often assemble into large irregular shape when the infection is advanced [35]. In this experiment, the herbaceous peony gray mold pathogen formed a large number of gathered sclerotia during the later growth stages, which was similar to the behavior of *B*. *cinerea*. Moreover, the host range of *B*. *paeoniae* is narrow; it reportedly only infects *Paeonia suffruticosa*, *Paeonia lactiflora*, *Hosta plantaginea*, *Polygonatum cyrtonema* and *Convallaria majalis* [16,47], while *B*. *cinerea* could infect a very large number of host plants, such as vegetables [6,8], fruits [1] and flowers [48]. This study showed that the host range of the pathogen was very extensive. In addition, a rDNA-ITS sequence analysis was also conducted, which showed that the pathogen examined herein was 99% similar to *B*. *cinerea*. In conclusion, the morphological characteristics and molecular data identified the pathogen in this study as *B*. *cinerea*.

### Changes of physiological indexes

When pathogens attack cells, they damage the structure and function of the membrane, leading to cell membrane permeability changes, electrolyte leakages and conductivity increases [49]. Many studies have shown that changes in the cell membrane permeability could reflect the disease resistance of the plant cultivars–smaller changes in the conductivity in response to infection indicate a stronger resistance of the cultivar [36,50]. In this study, we found that the relative conductivity of two cultivars increased over time; the conductivity of ‘Dafugui’ was consistently higher than that of ‘Zifengyu’. This phenomenon indicated that the ‘Dafugui’ cells suffered more from *B*. *cinerea* stress than ‘Zifengyu’ cells, which proved that the ‘Dafugui’ cultivar was less resistant to disease.

Chlorophyll is one of the most important plant photosynthetic pigments, and its content is markedly reduced after pathogen infection. Specifically, the reduction in chlorophyll is evident in disease-sensitive but not in disease-resistant cultivars [37,38,51]. This study also showed that the chlorophyll content was damaged in both cultivars after pathogen infection, but the impact was less in the ‘Zifengyu’ cultivar than in the ‘Dafugui’ cultivar, which further proved that ‘Zifengyu’ was more disease resistant. In summary, the relative conductivity and the content of chlorophyll were utilized as physiological indexes to assess the ability of herbaceous peony to resist gray mold.

### Defense response mechanisms of herbaceous peony in response to *B*. *cinerea-*induced stress

During long-term interactions with pathogens, plants develop a set of natural immune responses, including at least two types of defense reactions, namely PAMP-triggered immunity (PTI) and Effector- triggered immunity (ETI) [52,53]. During PTI, pathogen-associated molecular patterns (PAMPs) are recognized by pattern recognition receptors (PRRs) on the cell membrane of the plant, which induces a series of defense reactions in host plants, including the formation of phytoalexin (PA) and the expression of pathogenesis-related proteins (PRs) [54,55]. Because the effector proteins secreted by pathogens can inhibit PTI, plants have evolved another defense mechanism, ETI. During ETI, the plant resistance proteins (R proteins) recognize the pathogenic effector proteins, which causes a series of specific defense responses [53,56].

Receptor-like kinases (RLKs) are typical PRRs on the cell membrane [57] that play a key role in the detection of pathogen infection and signal transduction [6,58]. De Cremer et al. [6] identify several types of RLKs that are differentially expressed during lettuce / *B*. *cinerea* interactions. This phenomenon was similarly identified in herbaceous peony infected with *B*. *cinerea*. The largest family of PRRs included leucine-rich repeat RLKs (LRR-RLKs), some of which were significantly up-regulated in this study. Specifically, eleven *BAK1* genes, two *BRI1* genes, three *EFR* genes and seven *FLS2* genes were significantly up-regulated in the ‘Zifengyu’ cultivar during the early stages of infection, but they were gradually up-regulated in the ‘Dafugui’ cultivar during the same stage. Kemmerling et al. [59] show that BRI1 and BAK1 operate as co-receptors to control cell death by inhibiting bacterial infection. While Chinchilla et al. [60] provide evidence that BAK1 and FLS2 form a complex in a specific ligand-dependent manner to first detect pathogenic stimulation via flagellin; this detection positively regulates PRRs-dependent signaling to activate the innate immune system. Furthermore, a PRR CERK1 containing a lysin motif (LysM) domain that served as the receptor of chitin, a component of the fungal cell wall, was also identified in this study. Two genes that encoded CERK1 were both up-regulated as a whole in two cultivars. Studies have proved that the CERK1 in *A*. *thaliana* could directly combine with chitin to initiate a defense reaction [61], which also plays an important role in the disease resistance response during the *A*. *thaliana / P*. *syringa* interaction [62]. In addition, one *WRKY29* gene, five *WRKY33* genes and one *PR1* gene were up-regulated. WAKY29 and WRKY33 are members of the WRKY transcription factor family, which positively regulates the plant defense response [63,64]. The expression of PRs rapidly increases in the plant body after infection by a variety of pathogens [65]. PRs participate in resistance reactions by solidifying cell walls, enhancing the antibiosis activity or involving cell signal transduction [66,67]. PR1, which was identified in this experiment, was frequently cited as molecular marker of plant defense response activation [68]. Moreover, the expression levels of the screened *RBOH* and *CDPK* genes in ‘Dafugui’ were higher than those of ‘Zifengyu’ during each stage, which was completely different from other candidate disease resistant genes. It was well known that RBOH played a vital role in generating ROS, which could limit pathogen growth and facilitate cell death, thus inducing resistance [69]. And CDPK could reportedly improve the activity of RBOH to induce the generation of ROS [70,71]. However, the role of ROS in resistance was remarkably dependent on the life style of the pathogen. For plant cells, it was believed that the HR-associated cell death caused by sustained production of ROS may promote susceptibility to the necrotroph *B*. *cinerea*, while indeed ROS contributed to resistance at early stages of infection [72,73]. Meanwhile, the up-regulated genes *SOD*, *POD*, and *GST* were identified in the pathway related to the antioxidant system, which could timely scavenge ROS and prevent plant cells from damage. In the comparison of two cultivars, we found the expression levels of antioxidant enzyme in ‘Zifengyu’ visibly exceeded those of ‘Dafugui’. Overall, RBOH and CDPK were up-regulated after the invasion of *B*. *Cinerea* in two herbaceous peony cultivars in this study, thus generating ROS. The antioxidant enzyme of ‘Zifengyu’ were sharply induced at early phase to suppress the oxidative burst and thus resistant to the virulence of the necrotrophic pathogen. However, the expression level was relatively lower in ‘Dafugui’, which led to sustained production of ROS and aggravation of infection.

The resistance genes *PBS1*, *RPM1* and *MYC2* associated with ETI were also identified downstream of the “Plant-pathogen interaction” pathway. Serine/threonine-protein kinase PBS1 recognizes the effector protein AvrPphB of *pseudomonas syringae* and then participates in the ETI triggered by the effector, which leads to HR [74]. In plants, RPM1 interacting protein 4 (RIN4), RPM1 and RPS2 exist as a complex, and RIN4 acts as the inhibiting factor to maintain RPM1 and RPS2 in the inactive state. Avirulence genes activate RIN4 via the phosphorylation and degradation of RIN4 in response to infection, which activates the defense reaction mediated by RPM1 [75,76]. As an important member of jasmonic acid (JA) signaling pathway, transcription factor MYC2 initiates the expression of defense-relevant genes to induce defense reactions [77]. *PBS1*, *RPM1* and *MYC2* genes were all up-regulated in herbaceous peony in response to pathogen stress, which may be an important mechanism of disease resistance in herbaceous peony. The expression of disease resistance-relevant genes increased by several fold in ‘Zifengyu’ during the early stages of infection, which may confer this cultivar with strong disease resistance.

Secondary metabolites include phenols, terpenoids and nitrogen compounds [78], which could serve as biochemical barriers to withstand pathogen infection or act as signal substances in plant resistance reactions [39]. Among these substances, lignin is a type of phenolic polymer with a complex structure, which is an ingredient of the plant cell wall. When plant tissues are infected by pathogens, large amounts of lignin are observed to accumulate in the cell wall [79], which forms a structural and chemical barrier to restrict the invasion, proliferation, growth and reproduction of pathogens. In this study, we selected several genes related to the formation of lignin from “Phenylpropanoid biosynthesis” pathway, such as *4CL* and *HCT*, which controlled the biosynthesis of the G-lignin monomer and S-lignin monomer [80–82], *COMT* and *F5H*, which participated in the formation of the S-lignin monomer [83,84], and *UGT72E*, which catalyzed the sinapyl alcohol and coniferyl alcohol precursors of lignin biosynthesis [85]. The expression levels of these genes were up-regulated overall and increased with the infection time, but the multiple of expression levels of these genes were higher in the ‘Zifengyu’ cultivar than in the ‘Dafugui’ cultivar during the early stages of the infection. Phytoalexins are small molecule compounds with antimicrobial activity that are induced by pathogens in plants to activate or inhibit the synthesis of relevant enzyme genes, which form the chemical barrier to defend against pathogens [86]. We identified many phytoalexin synthesis-relevant genes in this experiment, primarily in the “Isoflavonoid biosynthesis”, “Diterpenoid biosynthesis”, “Sesquiterpenoid and triterpenoid biosynthesis” and “Terpenoid backbone biosynthesis” pathways. Moreover, the benzoxazinoid synthesis-related genes *Bx6*, *Bx7*, *Bx8* and *Bx9* were also screened, and the secondary metabolites formed by these genes all played important roles in the plant defense reactions against pathogens [87–89].

In general, the PRRs BAK1, BRI1, EFR, FLS2 and CERK1 on the cell membrane first recognized PAMPs in response to *B*. *cinerea* infection in herbaceous peony, which opened the calcium channels and then activated CDPK to release ROS. This process damaged the cell membrane, increased the relative conductivity and decreased the concentration of chlorophyll. A signal indicating that the cell was under attack was then passed, which activated the antioxidant enzymes SOD, POD, GST to scavenge ROS. The transcription factor WRKY and PR protein synthesis were up-regulated to initiate defense reactions. Secondary metabolites, such as lignin, phytoalexins and benzoxazinoids, were also induced to form biochemical barriers as part of the PTI. When the PTI was inhibited by pathogens, the up-regulation of disease resistance-relevant genes *PBS1*, *RPM1* and *MYC2* induced specific defense responses, namely ETI. Furthermore, because the pathogen recognition, the consequent defense reaction and the up-regulation of disease-resistance genes all occurred rapidly in the disease-resistant cultivar ‘Zifengyu’, this cultivar was more resistant to the disease than the ‘Dafugui’ cultivar. As a result, the relative conductivity increase and chlorophyll decrease were less pronounced in the ‘Zifengyu’ cultivar than in the ‘Dafugui’ cultivar.

## Supporting Information

S1 Fig Electrophoretic pattern of pathogenic rDNA-ITS sequence amplification. Marker is DL5000 DNA Marker. Water and Strain mean that ddH2O and the isolated strain DNA are added in the agarose gel electrophoresis detection, respectively. 1000bp, 750bp, 500bp and 250bp mean the band size of the marker.
(TIF)

S2 Fig Composition of raw reads of each library. S1: late May, S2: middle June, S3: early July, S4: late July.
(TIF)

S3 Fig Sequencing saturation of each library. S1: late May, S2: middle June, S3: early July, S4: late July.
(TIF)

S4 Fig Distribution of reads on reference genes of each library. S1: late May, S2: middle June, S3: early July, S4: late July.
(TIF)

S5 Fig DEGs identified in each library contrast of different cultivars at the same stage. S1: late May, S2: middle June, S3: early July, S4: late July. Red scatters indicate up-regulated DEGs, green scatters indicate down-regulated DEGs, and blue scatters indicate no difference DEGs in expression between the libraries.
(TIF)

S6 Fig Go classifications of DEGs in each library contrast of different cultivars at the same stage. S1: late May, S2: middle June, S3: early July, S4: late July. DEGs are annotated in three categories: biological process, cellular component and molecular function.
(TIF)

S1 Table Gene-specific primers sequence for qRT-PCR validation of DGE data. (XLS)

S2 Table Terms related to *B*. *cinerea*-induced stress from biological process ontology in ‘Zifengyu’-S1 vs. ‘Zifengyu’-S2. Data only showed the Gene Ontology terms that Corrected P-value ≤ 0.05.
(XLS)

S3 Table Terms related to *B*. *cinerea*-induced stress from biological process ontology in ‘Dafugui’-S1 vs. ‘Dafugui’-S4. Data only showed the Gene Ontology terms that Corrected P-value ≤ 0.05.
(XLS)

S4 Table Terms related to *B*. *cinerea*-induced stress from biological process ontology in ‘Zifengyu’-S4 vs. ‘Dafugui’-S4. Data only showed the Gene Ontology terms that Corrected P-value ≤ 0.05.
(XLS)

S5 Table KEGG pathway for ‘Zifengyu’-S1 vs. ‘Zifengyu’-S4. Data only showed the pathways that Corrected Q-value ≤ 0.05.
(XLS)

S6 Table KEGG pathway for ‘Dafugui’-S1 vs. ‘Dafugui’-S4. Data only showed the pathways that Corrected Q-value ≤ 0.05.
(XLS)

S7 Table The candidate DEGs involved in plant-pathogen interaction. (XLS)

S8 Table The candidate DEGs involved in secondary metabolism. (XLS)

S9 Table The candidate DEGs involved in antioxidant system. (XLS)
