# Supplementary figures and images for: Digital Gene Expression Analysis to Screen Disease Resistance-Relevant Genes from Leaves of Herbaceous Peony (Paeonia lactiflora Pall.) Infected by Botrytis cinerea
Source: PLoS One. 2015 Jul 24;10(7):e0133305. doi: 10.1371/journal.pone.0133305 (PMC4514867; doi:10.1371/journal.pone.0133305)

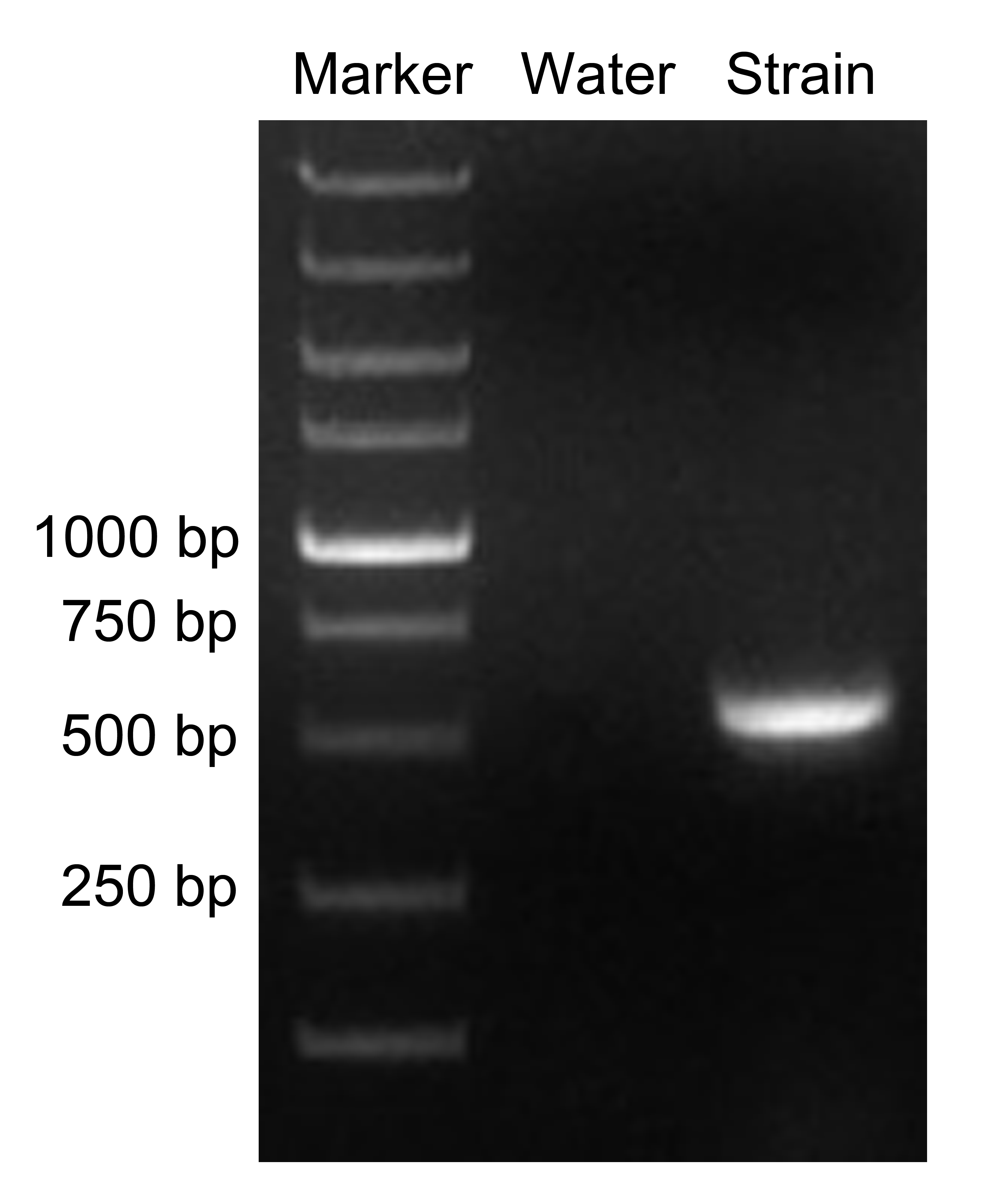

Supplement: S1 Fig — Marker is DL5000 DNA Marker. Water and Strain mean that ddH2O and the isolated strain DNA are added in the agarose gel electrophoresis detection, respectively. 1000bp, 750bp, 500bp and 250bp mean the band size of the marker. (TIF) [file pone.0133305.s001.tif]

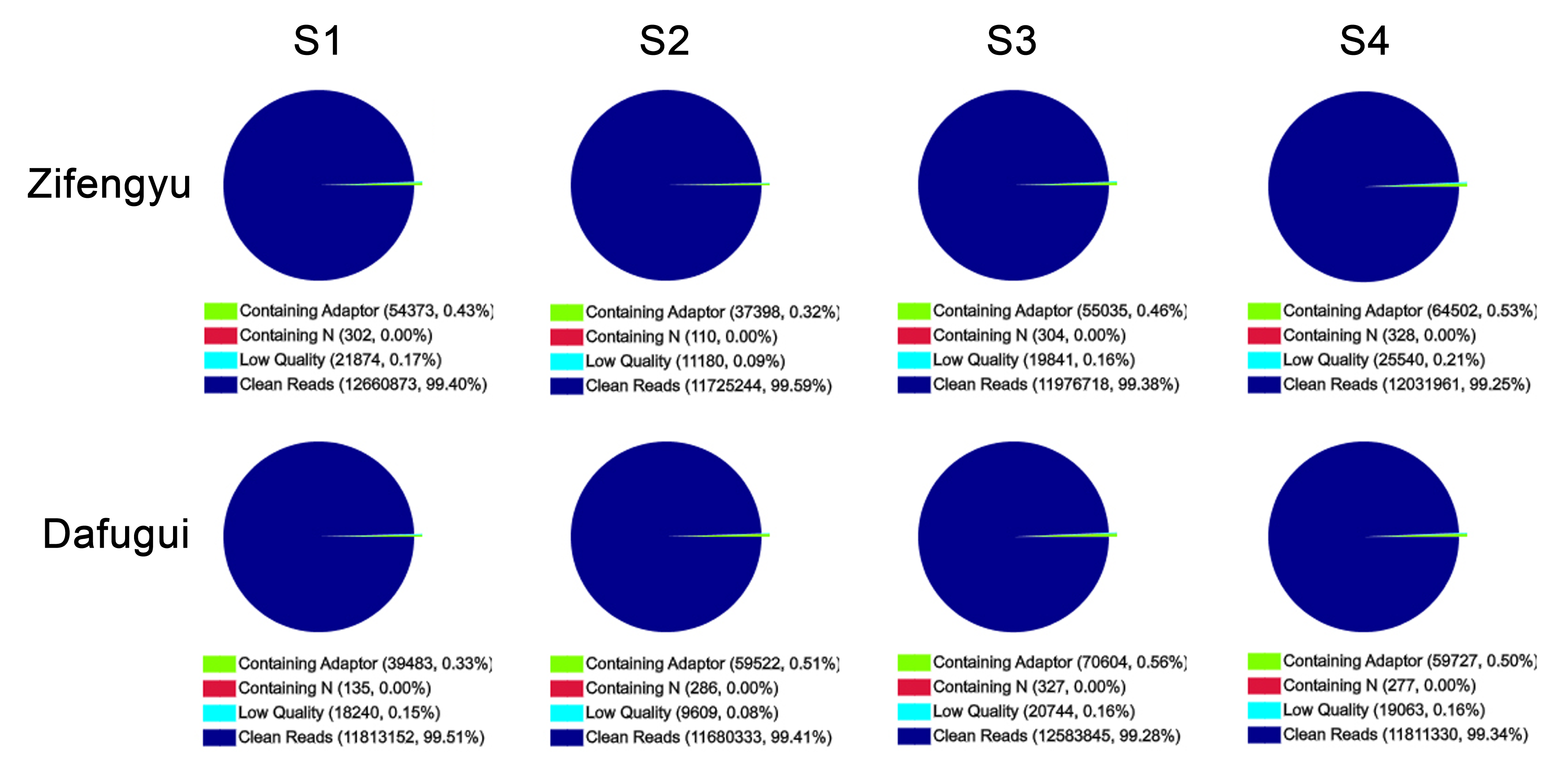

Supplement: S2 Fig — S1: late May, S2: middle June, S3: early July, S4: late July. (TIF) [file pone.0133305.s002.tif]

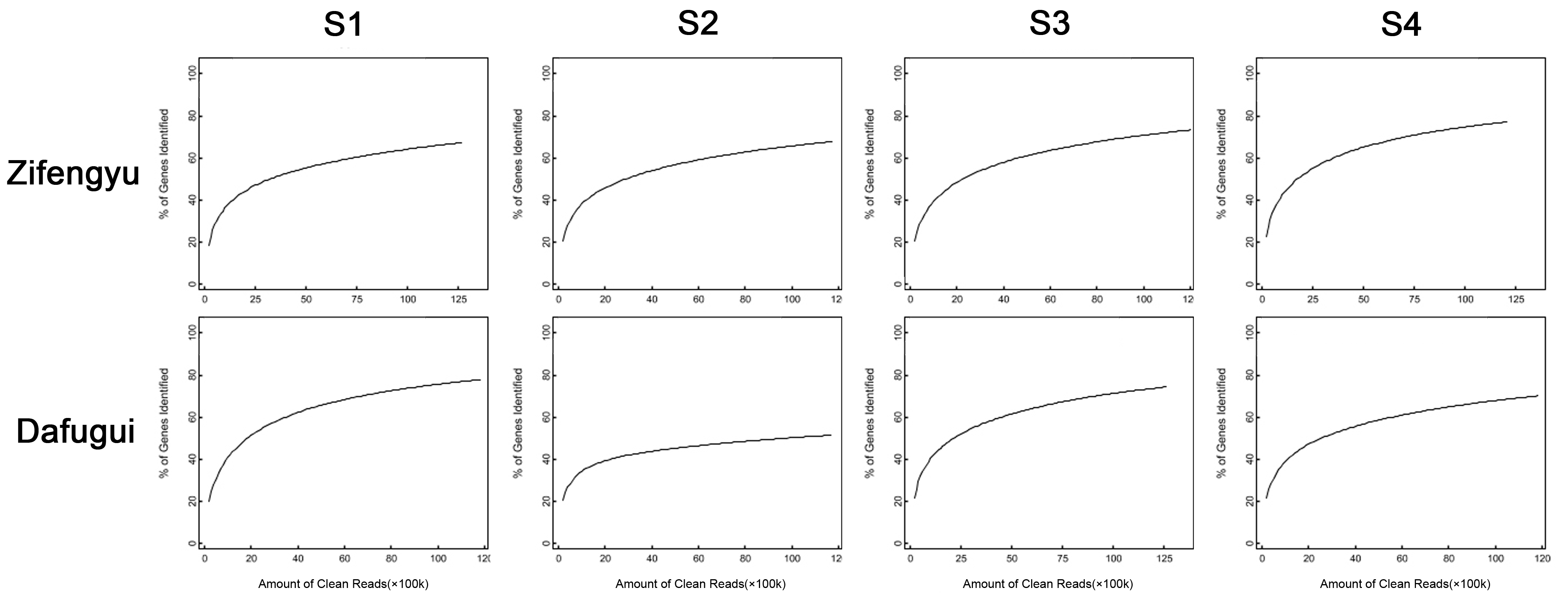

Supplement: S3 Fig — S1: late May, S2: middle June, S3: early July, S4: late July. (TIF) [file pone.0133305.s003.tif]

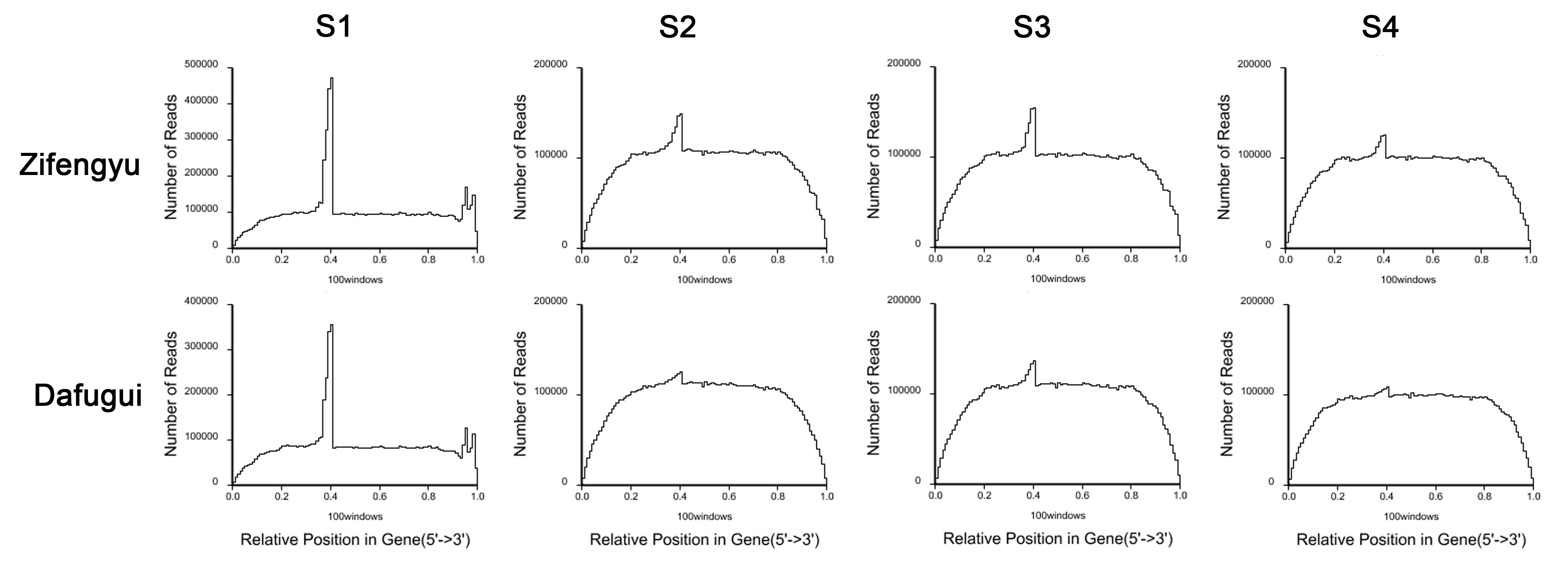

Supplement: S4 Fig — S1: late May, S2: middle June, S3: early July, S4: late July. (TIF) [file pone.0133305.s004.tif]

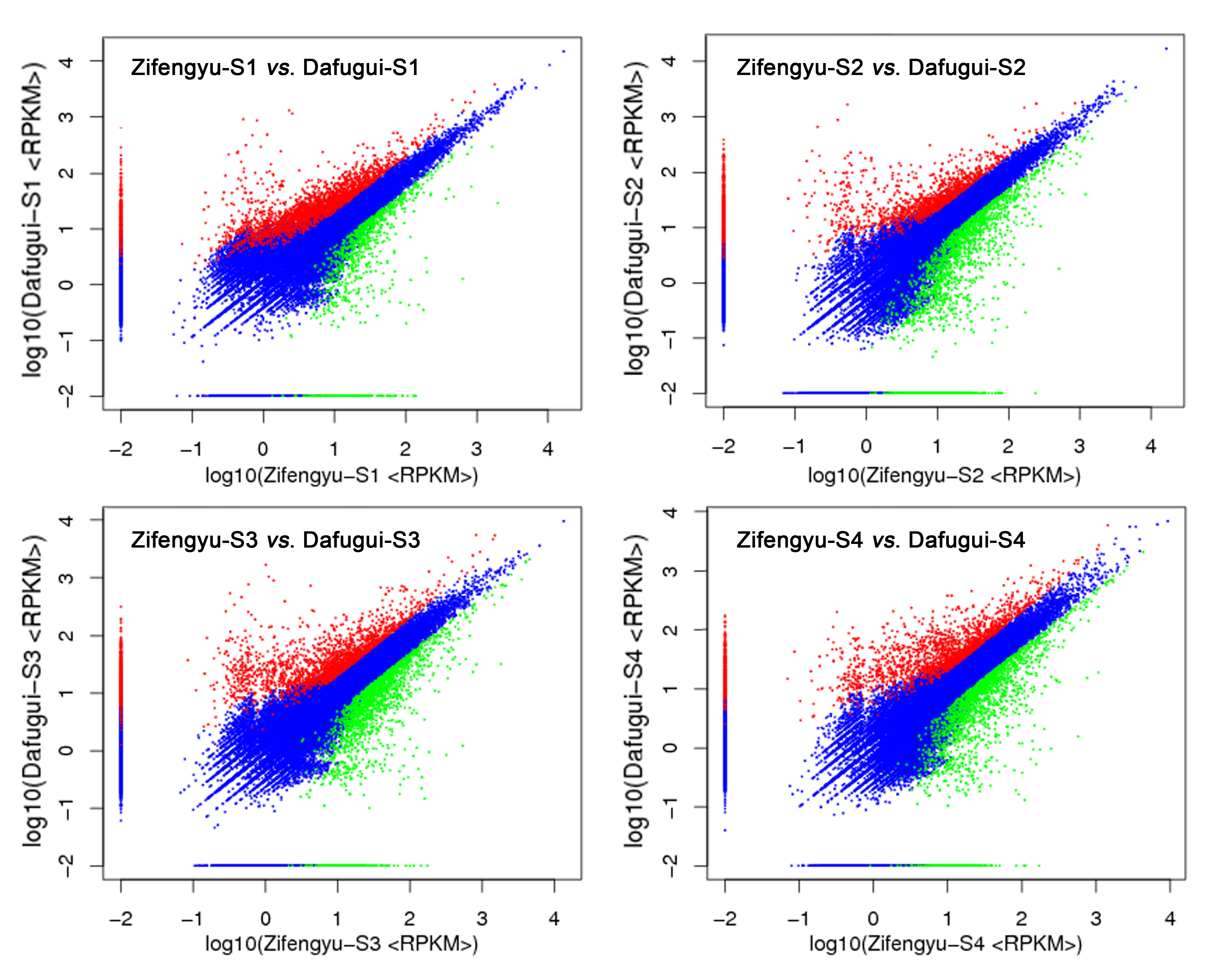

Supplement: S5 Fig — S1: late May, S2: middle June, S3: early July, S4: late July. Red scatters indicate up-regulated DEGs, green scatters indicate down-regulated DEGs, and blue scatters indicate no difference DEGs in expression between the libraries. (TIF) [file pone.0133305.s005.tif]

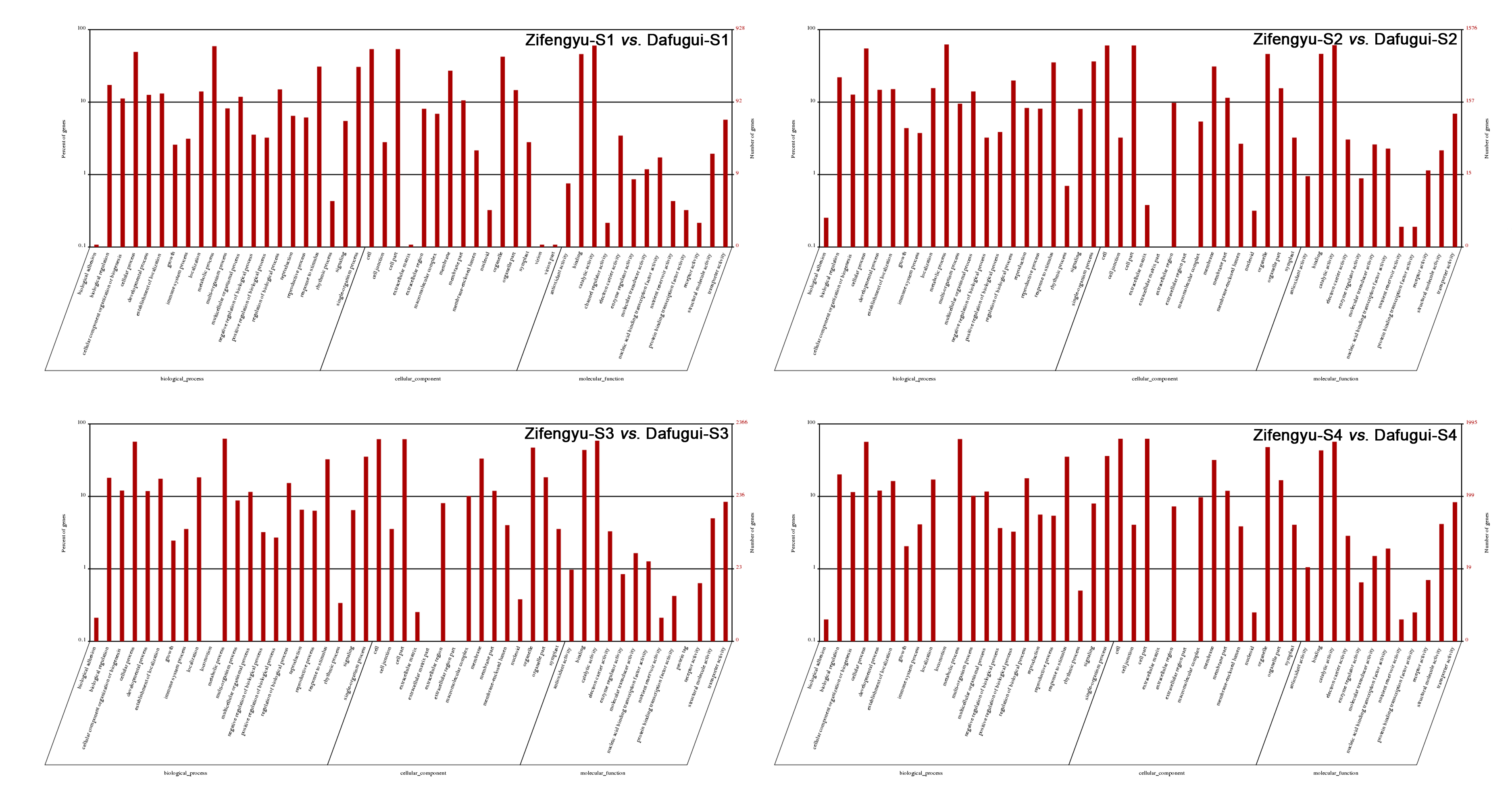

Supplement: S6 Fig — S1: late May, S2: middle June, S3: early July, S4: late July. DEGs are annotated in three categories: biological process, cellular component and molecular function. (TIF) [file pone.0133305.s006.tif]
